# Understanding the robustness of vision-language models to medical image artefacts

**DOI:** 10.1038/s41746-025-02108-w

**Published:** 2025-11-27

**Authors:** Zijie Cheng, Ariel Yuhan Ong, Siegfried K. Wagner, David A. Merle, Lie Ju, Hanyuan Zhang, Ruinian Chen, Linze Pang, Boxuan Li, Tiantian He, Anran Ran, Hongyang Jiang, Dawei Gabriel YANG, Ke Zou, Jocelyn Hui Lin Goh, Sahana Srinivasan, Andre Altmann, Daniel C. Alexander, Carol Y. Cheung, Yih Chung Tham, Pearse A. Keane, Yukun Zhou

**Affiliations:** 1 Department of Medical Physics & Biomedical Engineering, University College London, London, UK; 2 Institute of Ophthalmology, University College London, London, UK; 3 NIHR Biomedical Research Centre, Moorfields Eye Hospital NHS Foundation Trust, London, UK; 4 UCL Hawkes Institute, University College London, London, UK; 5 Department of Computer Science, University College London, London, UK; 6 School of Computer Science and Technology, Beijing Jiaotong University, Beijing, China; 7 Department of Ophthalmology and Visual Sciences, The Chinese University of Hong Kong, Hong Kong Special Administrative Region, Hong Kong, China; 8 Singapore Eye Research Institute, Singapore National Eye Centre, Singapore, Singapore; 9 Centre for Innovation and Precision Eye Health; and Department of Ophthalmology, Yong Loo Lin School of Medicine, National University of Singapore, Singapore, Singapore; 10 Ophthalmology and Visual Science Academic Clinical Program, Duke-NUS Medical School, Singapore, Singapore

**Keywords:** Computational biology and bioinformatics, Health care, Mathematics and computing, Medical research

## Abstract

Vision-language models (VLMs) show promise for answering clinically relevant questions, but their robustness to medical image artefacts remains unclear. We evaluated VLMs’ robustness through their performance on images with and without weak artefacts across five artefact categories, as well as their ability to detect images with strong artefacts. We built evaluation benchmarks using brain MRI scans, Chest X-ray, and retinal images, involving four real-world medical datasets. VLMs achieved moderate accuracy on original unaltered images (0.645, 0.602 and 0.604 for MRI, OCT, and X-ray applications, respectively). Accuracy declined with weak artefacts (−3.34%, −9.06% and −10.46%), while strong artefacts were detected at low rates (0.194, 0.128 and 0.115). Our findings indicated that VLMs are not yet capable of performing tasks on medical images with artefacts, underscoring the need to establish uniform benchmark thoroughly examining model robustness to image artefacts, and explicitly incorporate artefact-aware method design and robustness tests into VLM development.

## Introduction

Vision-language models (VLMs) are trained using vast and diverse images paired with textual descriptions (prompts), allowing them to deeply understand visual content and generate accurate responses^1^. Powerful examples, such as GPT-4o^2^ and Claude 3.5 Sonnet^3^, enable diverse applications such as image captioning^4^. The advances have been extended to the clinical task, leading to specialised medical VLMs such as MedDR^5^, LLaVAMed^6^ and BiomedCLIP^7^, which contribute to disease detection in multiple medical imaging modalities. However, in real-world medical scenarios, medical image artefacts commonly exist and potentially bias the performance of VLMs, which has not been systematically studied.

Image artefacts in the medical domain are distortions caused by patient movement, equipment limitations, varied technicians’ skills and environmental factors^8–12^. They are commonly seen in many medical imaging modalities such as magnetic resonance imaging (MRI)^13^, optical coherence tomography (OCT)^14^ and X-ray^15^. For instance, random motion in OCT imaging refers to movements of the subject, eye drift or microsaccades during scan acquisition^14,16,17^. These image artefacts can lead to errors in disease detection, including false positives, where normal cases are incorrectly identified as diseased, and false negatives, where diseased cases are mistakenly classified as normal^18–21^. These errors occur when random image artefacts generate patterns similar to pathological features or obscure actual abnormalities. Previous literature has studied the impact of medical image artefacts on traditional Artificial Intelligence (AI) models and have observed remarkable performance drops^22–24^.

**Fig. 1:**
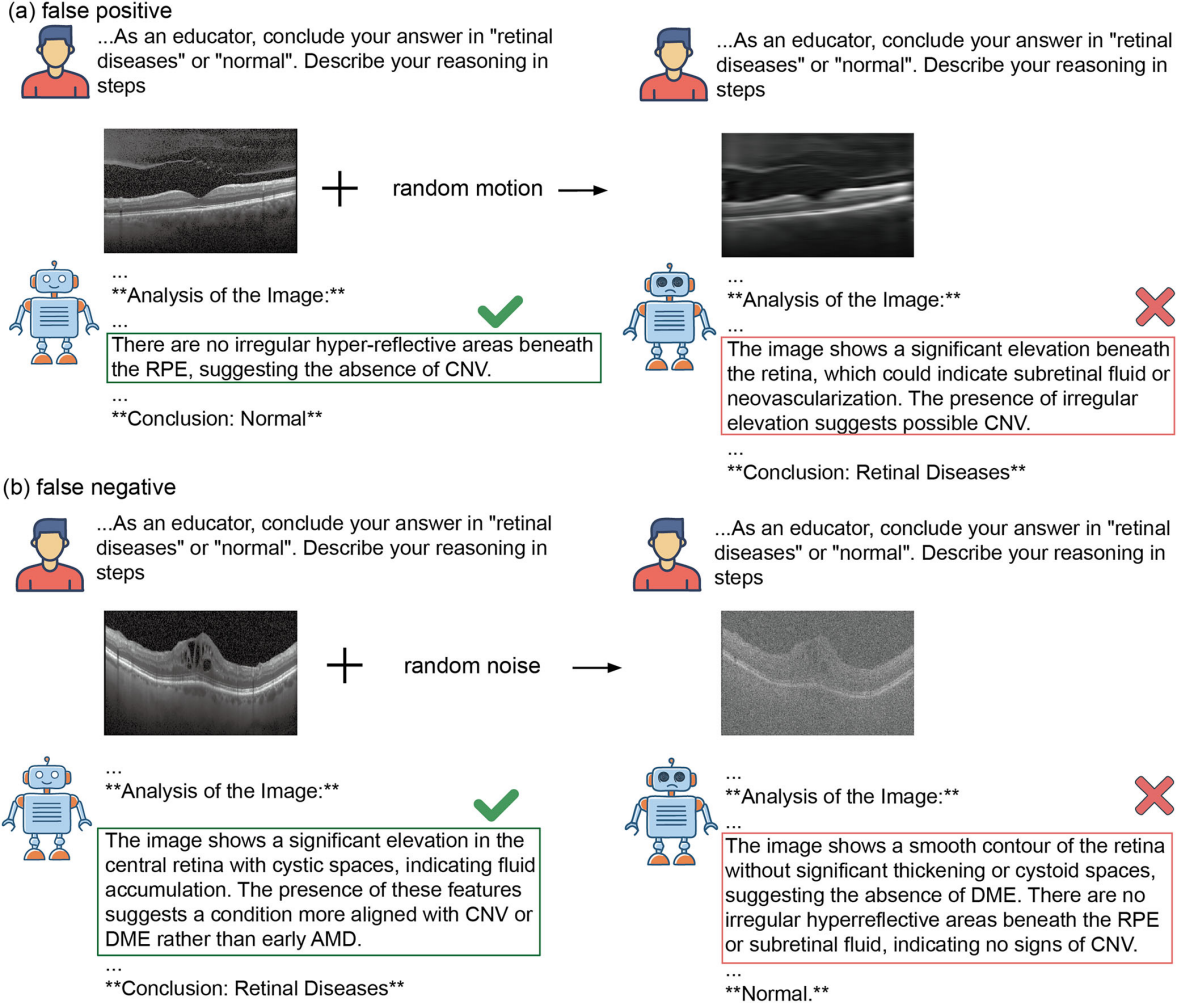
False positive and False negative examples after adding artefacts to images. **a** An example of a false positive case. Vision-Language models (VLMs) correctly classify normal cases when analysing original unaltered images but misclassify the same cases as diseased when random motion is introduced. **b** An example of a false negative case. VLMs correctly identify abnormal cases in original unaltered images but misclassify them as normal after random noise is introduced. More misdetection examples with full VLMs’ responses shown in Supplementary Data 1.

Recent studies have explored the use of VLMs in medical visual question answering (VQA) to evaluate their capabilities in disease detection^25–29^. Their results show that some powerful VLMs (e.g. GPT-4o) could achieve performance comparable to junior doctors^30–34^ in certain tasks. However, few studies have explored the robustness and detection capabilities of VLMs when medical image artefacts are present. Understanding this critical knowledge from the early stages is essential for advancing trustworthy medical AI applications in clinical settings, especially as VLMs are increasingly seen as promising solutions due to their strong capabilities in reasoning and user interaction. Figure 1 illustrates failure cases in disease detection by VLMs, caused by the presence of medical image artefacts.

In this study, we evaluated the robustness of VLM to medical image artefacts in disease detection, with three particular contributions: (1) we constructed an evaluation benchmark for VLM robustness, spanning three medical imaging modalities, including five categories of image artefacts, in weak and strong scales; (2) we proposed to evaluate the robustness in three tiers of metrics to assess VLMs’ performance, the typical classification performance (i.e. accuracy and sensitivity), performance percentage change, which describe the proportional change in VLMs’ performance when adding weak artefacts to images, and the strong artefact detection rate which measures the VLMs’ ability to identify images with strong artefacts; (3) we included most competitive VLMs, including GPT-4o, Claude 3.5 Sonnet, Llama 3.2, BiomedCLIP and MedGemma and assessed their robustness in both datasets with added artefacts and real-world artefacts. We revealed the insufficient robustness of current VLMs to medical image artefacts and underscore the urgent need to highlight robustness tests in VLM benchmarking and to build more robust medical VLMs for healthcare applications.

## Results

**Fig. 2:**
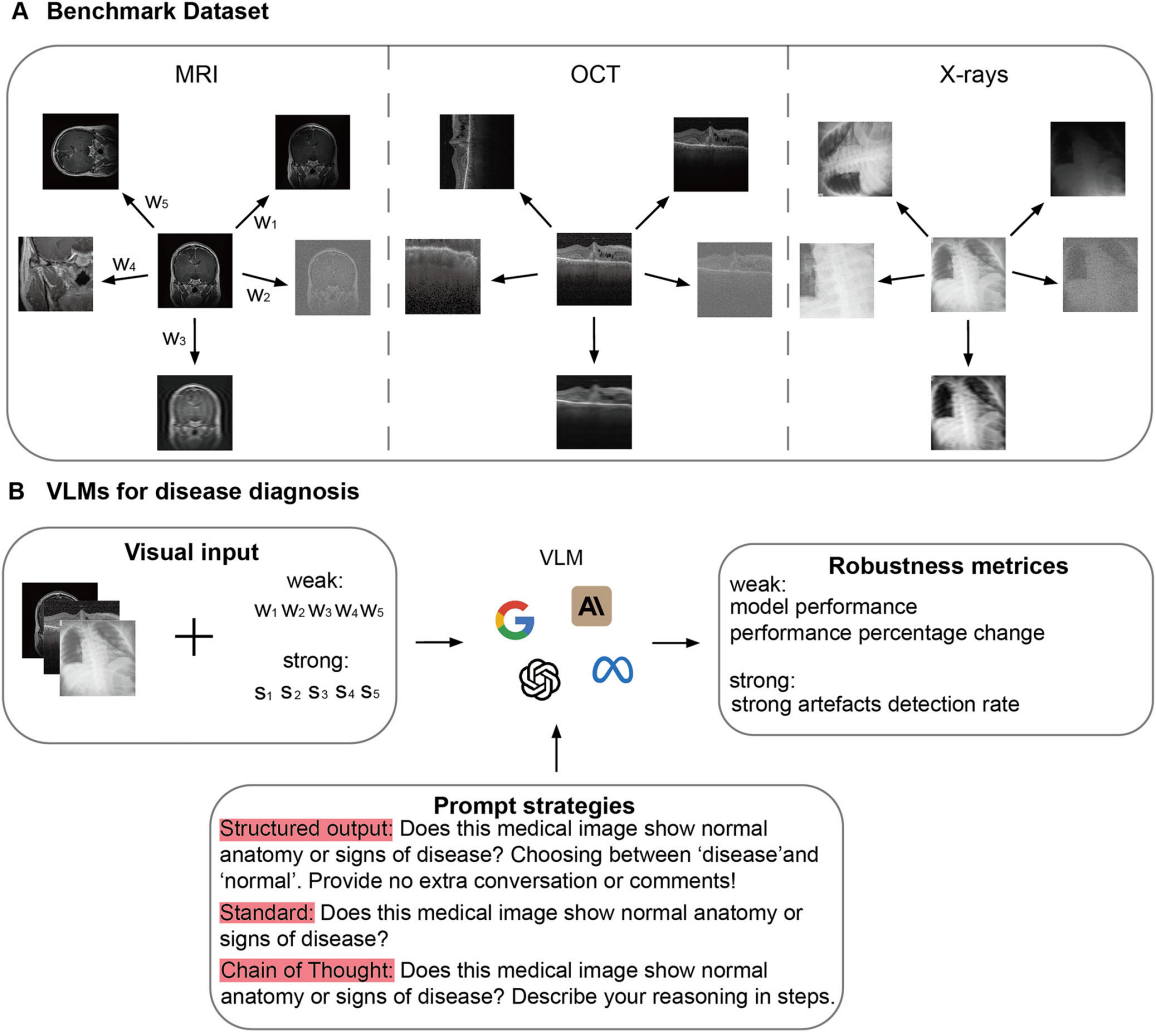
Overview of building benchmarks and the pipeline for evaluating robustness of Vision-Language models (VLMs) on disease detection tasks. **A** presents robustness evaluation benchmarks covering disease detection tasks across different medical imaging modalities. Three intensity artefacts (random bias field, noise and motion) with weak (w₁–w₃) and strong (s₁–s₃) scales, and two spatial artefacts (random cropping and rotation) with weak (w₄–w₅) and strong (s₄–s₅) scales are introduced to original unaltered images. All hyperparameters used to generate artefacts at weak and strong scales are provided in Supplementary Data 3. **B** illustrates the project pipeline, demonstrating the evaluation process used to assess the robustness of the VLMs. When adding weak artefacts, we evaluated model performance (e.g. accuracy) and the performance drop from its performance on the original unaltered images. When images were severely distorted by strong artefacts, we assessed VLMs’ ability to detect poor image quality. All experiments were repeated through different prompt strategies from restricting reasoning through structured output to encouraging reasoning step by step through Chain of Thought.

Figure 2 shows the overview of the project, including the construction of benchmarks and evaluation of VLMs’ robustness to medical image artefacts in disease detection tasks, spanning over three medical imaging modalities. For each medical imaging modality, we randomly selected 200 images, 100 for normal and 100 for disease cases. We built the benchmark by introducing common artefacts such as intensity artefacts (bias field, motion and noise) and spatial artefacts (cropping and rotation) to the original unaltered images at weak and strong scales. Weak artefact datasets consist of images that are partially obscured yet still interpretable, while strong artefact datasets contain ungradable images that are severely distorted by artefacts, making it impossible to reliably classify them as diseased or normal. More details are provided in the ‘Benchmark’ subsection of the Methods, with example images shown in Supplementary Figs. 1–3. The benchmark structure, along with the hyperparameters used in the TorchIO library to generate artefacts at weak and strong scales, is presented in Supplementary Data 2 and 3, respectively. This study included two proprietary models (GPT-4o^2^ and Claude 3.5 Sonnet^3^) and three open-source models (Llama 3.2^35^, BiomedCLIP^7^ and MedGemma^36^), with BiomedCLIP only output probability for pre-defined categories. We evaluated the typical classification performance of VLMs using original unaltered images and those containing weak artefacts. We also examined the performance percentage change after adding weak artefacts to images, as well as VLMs’ ability to detect strong artefacts (see the ‘Metrics for Model Robustness’ and ‘Models Evaluated’ subsections of the Methods section for further details). Additionally, we evaluated three prompting strategies: structured output, standard output and chain of thought, and examined their effect on performance. *p* values were calculated using a two-sided *t*-test to assess whether significant differences exist.

### Model performance on original unaltered images

**Fig. 3:**
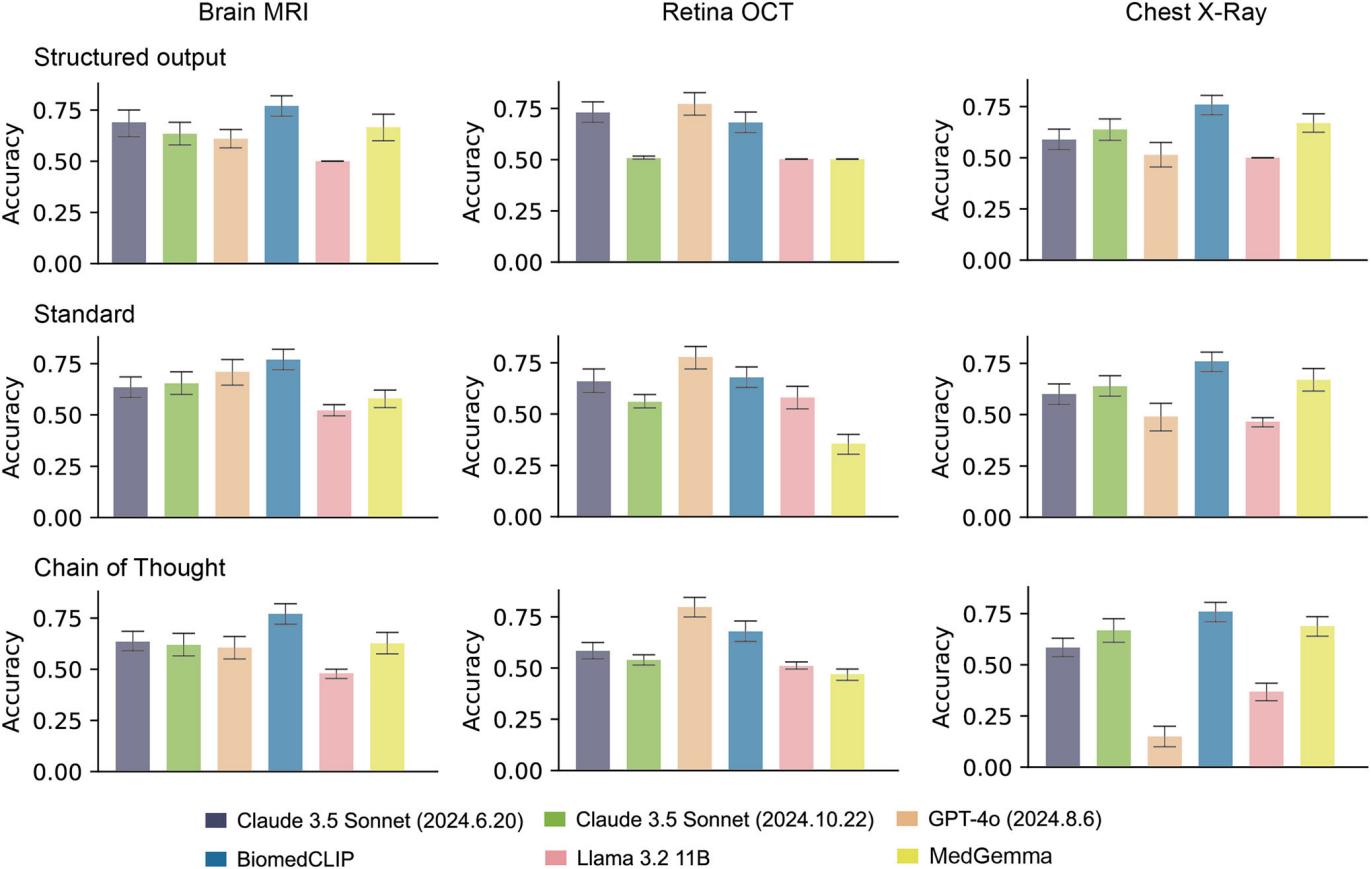
The accuracy of Vision-Language models (VLMs) in detecting disease on original unaltered images. Each column represents the models’ accuracy in disease detection tasks across different medical imaging modalities, while each row illustrates their accuracy using various prompt strategies. Quantitative results are detailed in Supplementary Data 6. For each task, accuracy measurements derive from 1000 stratified bootstrap samples. Results show mean accuracy with 95% confidence intervals (error bars).

We assessed the performance of various VLMs on original unaltered images and observed moderate performance (Fig. 3). For brain tumour (MRI) and COVID-19/pneumonia detection (chest X-ray), BiomedCLIP achieved the highest accuracy, with scores of 0.770 (95% Confidence Interval (CI): 0.720–0.820) and 0.760 (95% CI: 0.710–0.805) respectively, using the standard prompt. This advantage also extended to sensitivity, particularly in MRI applications, where it reached the highest value of 0.950 (95% CI: 0.910–0.990) (Supplementary Fig. 4). In macular disease detection (OCT), GPT-4o achieved the highest accuracy of 0.778 (95% CI: 0.720–0.830) and sensitivity of 0.656 (95% CI: 0.560–0.740) using the standard prompt. Llama 3.2 11B classified most cases as diseased, resulting in poor accuracy below 0.6 across all tasks (Fig. 3). In contrast, MedGemma classified most cases as normal, leading to similarly poor accuracy, high specificity, but low sensitivity (Fig. 3 and Supplementary Figs. 4, 5).

### Model robustness to weak image artefacts

**Fig. 4:**
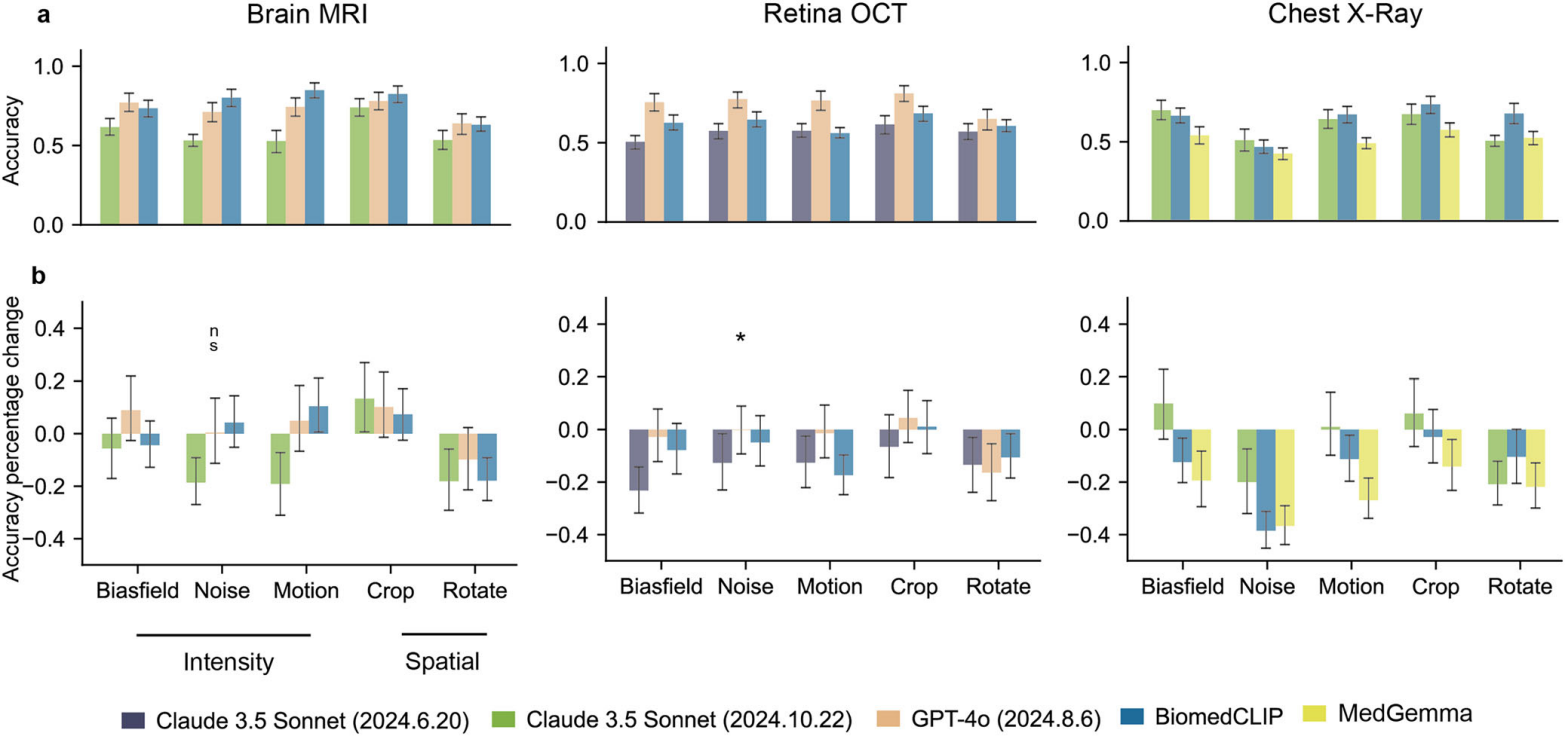
Accuracy of Vision-Language models (VLMs) detecting diseases in images with weak artefacts using standard prompts, and their performance percentage change compared to the accuracy obtained from original unaltered images. We present only the top three models for lesion detection on original unaltered images because the remaining models performed poorly, with accuracies around 0.5 (Fig. 3), and their performance on distorted images provides limited insight into model robustness. **a** Shows the models’ accuracy (y-axis) after adding weak artefacts to the images. Full models performance through various prompt strategies shown in Supplementary Figs. 6–8. **b** Illustrates the percentage change in models’ accuracy (y-axis) after introducing weak artefacts. Positive values indicate increased performance, whereas negative values represent decreased performance. *P* values comparing original model performance with performance after adding weak artefacts are shown above each bar. Unless otherwise specified, *p* < 0.001. ** indicates 0.001 < *p* < 0.01, * indicates 0.01 < *p* < 0.05 and ns indicates *p* ≥ 0.05. Model performance percentage change through various prompt strategies shown in Supplementary Figs. 9–17. The complete quantitative results are presented in Supplementary Data 7, while all *p* values can be found in Supplementary Data 9. For each disease detection task, we performed 1000 iterations of stratified bootstrapping to calculate accuracy and sensitivity. Results show mean performance with 95% confidence intervals (error bars).

We evaluated the robustness of VLMs on images with weak artefacts in disease detection (Fig. 4 and Supplementary Figs. 6–17). Figure 4a shows the top three models per type of disease detection task, selected based on their accuracy on original unaltered images (Fig. 3). Among the models evaluated, BiomedCLIP demonstrated the highest accuracy and sensitivity in MRI applications. For instance, it achieved an accuracy of 0.801 (95% CI: 0.745–0.855) and sensitivity of 0.782 (95% CI: 0.700–0.860) when detecting MRI images with weak random noise using the standard prompt (Supplementary Fig. 6). While in OCT applications, GPT-4o demonstrated the strongest performance (Supplementary Fig. 7). In chest X-ray applications, MedGemma consistently showed the lowest accuracy in Fig. 4a and the lowest sensitivity compared to all other models (Supplementary Fig. 8). We observed that MedGemma’s reasoning process is inconsistent with the conclusion in some cases. The reasoning process refers to the intermediate information provided by VLMs in analysing the provided image, while the conclusion summarises the VLM final decision, either ‘Normal’ or ‘Diseased’ in our study. We quantified the rate of such conflicts in Supplementary Fig. 19. For example, in chest X-ray applications using CoT prompting, MedGemma exhibited a conflict rate of 0.204 (95% CI: 0.150–0.260) on original images, which increased to 0.427 (95% CI: 0.360–0.490) after introducing weak random motion. Figure 4b shows the models’ performance percentage change relative to their original accuracy. Random noise resulted in more significant performance degradation, especially in X-ray applications. When random noise was added to chest X-rays, BiomedCLIP’s accuracy in detecting lung diseases decreased to 0.466 (95% CI: 0.425–0.510), representing a 38.5% drop (*p* < 0.001) compared to its performance on original unaltered chest X-rays.

We found that adding specific image artefacts improved the disease detection accuracy of certain VLMs, particularly in MRI applications. Specifically, applying weak random cropping to MRI images improved model performance. For instance, Claude 3.5 Sonnet (2024.10.22) achieved an accuracy of 0.739 (95% CI: 0.685–0.795) in detecting brain tumours, representing a 13.3% (*p* < 0.001) increase compared to the accuracy on original unaltered MRI images (Supplementary Data 8). Furthermore, introducing other image artefacts, such as the bias field artefact, to brain MRI images led to an 8.9% (*p* < 0.001) increase in brain tumour detection accuracy for GPT-4o.

### Strong artefacts detection rate

**Fig. 5:**
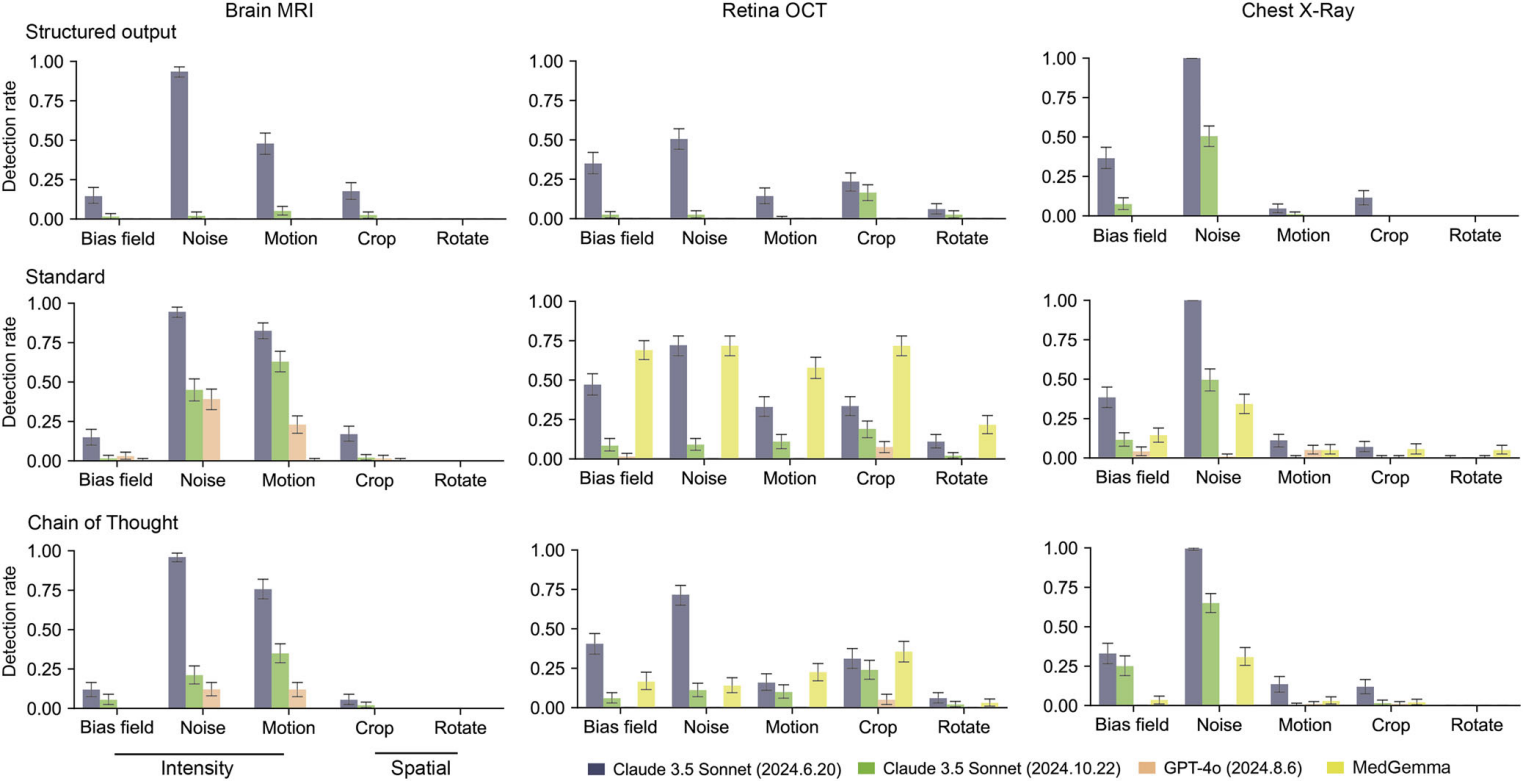
Vision-Language models’ (VLMs) detection rate for strong artefacts. The y-axis shows the rate at which VLMs flag poor image quality in images distorted by strong artefacts, with higher values indicating greater effectiveness in artefact identification. Quantitative results are presented in Supplementary Data 10. For each disease detection task, we performed 1000 iterations of stratified bootstrapping to calculate strong artefacts detection rate. Results show mean detection rates with 95% confidence intervals (error bars).

We assessed the VLMs’ ability to detect strong artefacts in severely distorted images (Fig. 5). We excluded BiomedCLIP, as it provides only category probabilities, i.e. disease or normal. Llama 3.2 11B was not included as it consistently classified most cases as diseased (Fig. 3 and Supplementary Figs. 4, 5). Claude 3.5 Sonnet (2024.6.20) with the standard prompt achieved the highest strong artefacts detection rate in MRI and X-ray applications. For example, on brain MRI images with strong random motion, Claude 3.5 Sonnet (2024.6.20) achieved a detection rate of 0.825 (95% CI: 0.775–0.875), outperforming both its upgraded model (0.629; 95% CI: 0.565–0.695) and GPT-4o (0.230; 95% CI: 0.175–0.285). MedGemma showed relatively good capability in detecting strong artefacts in OCT applications compared to other models. Specifically, it achieved a detection rate of 0.690 (95% CI: 0.630–0.750) on OCT images containing random bias fields, while Claude 3.5 Sonnet (2024.6.20) reached 0.471 (95% CI: 0.405–0.540). Intensity artefacts were generally more easily detected by VLMs. For instance, Claude 3.5 Sonnet (2024.6.20) achieved a 0.945 (95% CI: 0.910–0.975) detection rate when analysing MRI images with strong random noise, while showing no detecting capability for images with strong rotation.

### Role of prompt engineering

We analysed the impact of prompt engineering on VLMs’ performance. When switching prompt strategies from requiring structured output to standard output, models became more capable of flagging poor quality images. For instance, in MRI applications, Claude 3.5 Sonnet (2024.10.22) performed better with the standard prompt for detecting images with random noise, with rate of 0.629 (95% CI: 0.565–0.695), compared to 0.051 (95% CI: 0.025–0.080) with the structured output prompt, as shown in Fig. 5. We also observed some unexpected findings. When detecting chest X-rays with weak artefacts, GPT-4o showed decreased accuracy and sensitivity after switching prompt strategies from requiring structured output to standard and CoT output. For example, its sensitivity decreased from 0.809 (95% CI: 0.720–0.880) with the structured output prompt to 0.331 (95% CI: 0.240–0.420) with the CoT prompt when detecting chest X-rays containing random motion artefacts, as shown in Supplementary Fig. 4.

### VLM robustness to real-world artefacts in applications with colour fundus photographs

We assessed VLM robustness in detecting diabetic retinopathy from colour fundus photographs containing real‑world artefacts of varying scales (Supplementary Fig. 18). Weak artefact datasets contain images that are partially obscured yet still interpretable, whereas strong artefact datasets contain ungradable images that are images severely distorted and without clearly visible lesions. The first two subsets each include two categories, normal and diabetic retinopathy, with 50 images per category. The third subset contains severely distorted and ungradable images. When detecting high quality images, GPT-4o had the highest accuracy 0.731 (95% CI: 0.660–0.800) and highest specificity 0.960 (95% CI: 0.900–1.000). While Llama 3.2 11B had the lowest accuracy 0.470 (95% CI: 0.430–0.500), as it classified most cases as diabetic retinopathy. When detecting images with weak artefacts, most models had a drop in accuracy. Specifically, MedGemma demonstrated the weakest robustness to image artefacts, exhibiting the largest drops in performance: a 41% decrease in accuracy and a 77.5% decrease in specificity compared to its performance on images without artefacts. Similar to the findings observed in X-ray applications, Claude series models exhibited increased sensitivity after introducing weak artefacts; however, this did not result in improved accuracy. In detecting ungradable images, MedGemma achieved the highest detection rate for poor quality images, at 0.780 (95% CI: 0.700–0.860).

## Discussion

This study presents a robustness analysis of the VLMs to medical image artefacts in disease detection. We developed an evaluation benchmark and proposed to evaluate model robustness in three tier metrics. The results indicate that all VLMs perform moderately on original unaltered images, and significantly worse when weak artefacts were added. Few VLMs were able to recognise the poor quality images, showing low strong artefact detection rate. We observed a similar VLM robustness on a dataset with real-world artefacts, further revealing the limited capability of current VLMs in handling images with suboptimal quality.

VLMs do not perform well on disease detection tasks and perform even worse when artefacts are added to the original unaltered images. Prior work reports higher performance for GPT-4o on selected disease detection tasks^25,27,28,37^. In this study, BiomedCLIP in certain applications, such as disease detection with chest X-ray (Fig. 3) performed well, surpassing competitive and multifold larger VLMs. Previous research suggests that models pre-trained on medical data benefits certain domain-specific tasks^38,39^, which likely explains the good performance of BiomedCLIP of certain medical applications. This highlights the value of domain-specific pre-training for downstream clinical applications like disease detection. This is further evidenced by the poor performance of MedGemma in OCT applications compared to applications in other medical imaging modalities, as it has not been specifically fine-tuned for this modality^36^. Part of our observations are consistent with past literature. Xia et al. investigated the robustness of VLMs to random noise and revealed that VLMs perform poorly under such condition^27^. More specifically, VLMs show poorer robustness against intensity artefacts, particularly random noise. This may be because spatial artefacts preserve more features compared to intensity artefacts (Supplementary Figs. 1–3). Additionally, despite image rotation only altering its orientation, this leads to performance degradation across all applications in our study, probably due to these models mostly trained on single-orientation images. In applications involving X-rays and colour fundus photographs with weak artefacts, Claude series models showed reduced accuracy but increased sensitivity, likely due to misinterpreting artefacts as lesions.

Few models are capable of flagging strong image artefacts in disease detection tasks. In recent studies, images in disease detection tasks are usually taken from public datasets with good image quality, without evaluating VLMs’ performance in detecting the poor image quality^13,15,16,40,41^. In this study, we find that Claude 3.5 Sonnet (2024.6.20) demonstrates superior performance in detecting strong artefacts compared to other VLMs. Understanding the rationale behind this is important but challenging, as these are proprietary models with undisclosed training data and strategies, which further emphasises the need for data transparency in VLM research^42,43^. Nevertheless, our evaluation pipeline provides a comprehensive comparison of their robustness to medical image artefacts. With the advancement of more powerful open-source VLMs, we plan to evaluate their robustness to image artefacts and provide deeper insights into the factors driving model performance. We also observe that VLMs are better at flagging intensity artefacts. This is probably because intensity artefacts largely change the intensity distribution compared to spatial artefacts (e.g. rotation). In the intensity artefacts, we find that VLMs are better at identifying severely distorted images caused by random noise (Fig. 5), likely due to their ability to recognise Gaussian distributions in image intensity distribution patterns. In contrast, other intensity artefacts such as the random bias field, which do not follow Gaussian distributions, present greater detection challenges for VLMs and require enhanced processing methods.

The variation in VLMs’ disease detection performance after adding weak image artefacts likely involves multiple factors. As shown in Fig. 4, the brain tumour detection performance for some VLMs was enhanced by introducing random cropping to MRI images. This improvement is likely due to cropping enabling the models to better focus on the anatomical tissue and lesion by removing extraneous blank background, with an example illustrated in Supplementary Fig. 21. We also observed that adding bias fields to brain MRI images improved the brain tumour detection accuracy for GPT-4o. This may be caused by varied selections in answered questions due to ethical safeguards within VLMs. When weak artefacts were added, some VLMs answered more questions than on the original images, while this didn’t extend to Chest X-ray and retinal applications. Future work will investigate the reasons behind the variation in VLMs’ performance after introducing certain types of image artefacts.

In certain cases, MedGemma’s final output diverges from the reasoning it presents, with an example of this discrepancy shown in Supplementary Data 13. This conflict was not observed in other models, possibly because MedGemma has a smaller size, with limited response generation capability. We also observed that as the scale of artefacts increased, the conflict rate of MedGemma rose correspondingly (Supplementary Fig. 19). Notably, MedGemma exhibited a higher rate of conflict with intensity artefacts compared to spatial artefacts. This may suggest that images with greater deviations from the normal intensity distribution cause more confusion for the model, indicating its limited robustness to medical image artefacts.

VLMs show inconsistent disease detection performance across medical imaging modalities. In OCT applications, VLMs show higher specificity but lower sensitivity compared to other medical imaging modalities. This may be attributed to the smaller size of lesions in OCT images, which are less distinguishable from normal tissue, leading to lower sensitivity. When detecting lesions on images with weak artefacts, VLMs show reduced sensitivity in MRI and OCT applications but increased sensitivity in X-rays. This paradox may stem from opacity being a key marker for pulmonary lesions—since healthy lungs appear with low opacity, artefacts that increase opacity may bias VLMs toward diseased predictions (Supplementary Fig. 3), raising sensitivity. In MRI applications, VLM performance typically improves with random cropping, whereas this effect is less evident in applications in other medical imaging modalities. This is likely because brain lesions are generally larger and centrally located, allowing cropping to help the model focus on the lesion (Supplementary Fig. 1d).

Allowing the reasoning process improves VLMs’ performance in recognising poor image quality, although it brings limited benefit to disease detection performance in general. Previous works report that CoT prompting can improve the VLMs’ performance on general tasks, particular tasks relying on logical thinking^44–48^. Whether this advantage extends to the medical domain, specifically in disease detection, has not been fully tested. In this study, we found that models with standard and CoT prompts outperformed structured output prompts in recognising poor image quality, possibly because they encourage models to focus more on the image itself. However, switching the prompt from structured output to standard or CoT formats could deteriorate the performance of some VLMs, particularly GPT-4o in X-ray applications (Fig. 3 and Supplementary Figs. 4, 5). This decline is caused by ethical safeguards of GPT-4o, refusing to answer more frequently with CoT prompts compared to structured output prompts. The ambiguous or refusal responses were categorised as incorrect answers in our study, as they provide limited valuable information to disease detection in clinical deployment. This choice has been widely used in other AI healthcare studies^25,27,49,50^. However, we acknowledge that this approach may underestimate the performance of certain VLMs in disease detection, particularly for models strongly constrained by ethical safeguards (e.g. GPT-4o on chest X-ray applications). The refusal or ambiguous outputs may reflect responsible model behaviour rather than an inability to perform the task. In the future, a benchmark considering both model performance and ethical safeguards should be designed for more comprehensive VLM evaluation.

A comprehensive evaluation of VLMs’ robustness is essential for advancing their development and explainability. Current models fall short in identifying poor-quality images. Future VLMs could benefit from integrating a quality assessment module prior to diagnosis. Moreover, our results may encourage researchers to investigate the underlying causes of VLMs’ suboptimal performance. For example, future work could visualise the attention maps from vision encoders to assess whether meaningful pathological features are captured across different medical images, particularly those with small lesion sizes (e.g. retinal OCT images in our study). Although the advantages of CoT reasoning may not be immediately apparent in current applications, prompt engineering remains a promising approach for enhancing disease detection performance, as shown in previous studies. Ferber et al. showed that in-context learning improves VLMs’ classification of cancer pathology images50. More broadly, systematic exploration of advanced prompting strategies may lead to further performance gains.

By building the VLMs’ robustness evaluation framework, we hope to accelerate the progress to better understanding and developing robust medical VLMs. Several limitations of this study warrant consideration. First, we introduced five categories of image artefacts commonly encountered in clinical scenarios. However, these may not fully capture the complexity of artefacts found in real-world medical imaging, where artefacts often appear in combination rather than isolation. It is worth exploring model robustness to combinations of image artefacts. Second, the intensity levels for weak and strong image artefacts were preset hyperparameters. Future research should provide a quantified threshold for weak and strong artefacts (e.g. quantifying the difference between images with weak artefacts and their original unaltered counterparts), therefore a more fine-grained scale of image artefacts will be explored. Third, disease detection tasks are generally simplified into a two-category format (normal vs. diseased), which is relevant to the moderate performance of VLMs even in these simplified cases (Fig. 3). Future work will further refine the VLM robustness evaluation framework by simulating more realistic image artefacts, exploring fine-grained artefact scales and incorporating multi-class disease classification tasks. Additionally, a detailed quantitative analysis of the effects of certain image artefacts that produced varied performance shifts will be undertaken to better understand their impact on VLM performance.

In this study, we build a benchmarking framework to evaluate VLMs’ robustness to image artefacts in three disease detection tasks across multiple medical imaging modalities. By pinpointing vulnerabilities tied to specific medical imaging modality and artefact types, this work provides insights for developing robust medical VLMs capable of functioning reliably in real-world clinical settings.

## Methods

### Benchmark dataset

The Brain Tumour MRI Dataset^51^ includes images of three tumour types—gliomas, meningiomas and pituitary tumours—along with healthy scans. It combines three sources: Figshare^52^, the SARTAJ dataset^53^, and Br35H^54^. Images in Figshare were acquired post-Gd-DTPA injection at Nanfang Hospital and Tianjin Medical University Hospital (2005.9–2010.10); the other sources lack detailed provenance. Patient age and sex are not provided. Each MRI scan yields multiple images, labelled by experienced radiologists. The study sample comprised 100 diseased and 100 normal images.

The Retinal OCT Images dataset^55^ includes three macular diseases—choroidal neovascularisation, diabetic macular oedema and drusen—alongside normal images. Images were collected from retrospective adult cohorts at five global institutions between July 1, 2013, and March 1, 2017. Patient age and sex are not provided. Multiple images per scan were labelled through a tiered grading process involving medical students, ophthalmologists and senior retinal specialists with over 20 years of experience. The study sample comprised 100 diseased and 100 normal images.

The COVID-19 Detection X-ray Dataset^56^ includes three lung diseases—bacterial pneumonia, COVID-19 and viral pneumonia—alongside normal chest X-rays. X-rays with COVID-19 were sourced from public datasets^54^, while pneumonia and normal were from paediatric patients (ages 1–5) at Guangzhou Women and Children’s Medical Centre^57^. Patient age, sex and number of scans per patient are not specified. Diagnoses were reviewed by two expert physicians. The study sample comprised 100 diseased and 100 normal images.

The benchmark consists of original unaltered images and those with artificially introduced artefacts at weak and strong scales (Supplementary Data 2). Weak artefact datasets consist of images that are partially obscured yet still interpretable, while strong artefact datasets contain ungradable images that are severely distorted by artefacts, making it impossible to reliably classify them as diseased or normal. Intensity artefacts (random noise, motion, bias fields) simulate issues from hardware noise and operational factors, applied using the TorchIO library^58^. Spatial artefacts (rotation, cropping) mimic misalignment and limited field of view during acquisition (Supplementary Figs. 1–3). The configuration and hyperparameter settings for all types of image artefacts at both weak and strong scales are provided in Supplementary Data 3. Questions were generated from ground truth labels to convert it into VQA tasks. This study did not involve the recruitment of human participants. All analyses were performed on publicly available, de-identified datasets (e.g. The Retinal OCT Images dataset^55^), with their details and links introduced in the ‘Data availability’ section. Additional ethical approval and informed consent were not required for this study.

To better assess the robustness of VLMs to compound artefacts existing in real-world data, we included DDR dataset, which contains diabetic retinopathy images of varying quality. Examples of image with compound artefacts are shown in Supplementary Fig. 20^59^. Collected from 147 hospitals across 23 provinces in China, the dataset lacks details on patient age, sex and scan count. Images were annotated by ophthalmologists and classified into six DR severity levels: none (‘0’), mild non-proliferative (‘1’), moderate non-proliferative (‘2’), severe non-proliferative (‘3’), proliferative (‘4’) and ungradable (‘5’). Ungradable images are those that are so severely distorted by artefacts that they cannot be reliably classified as diseased or normal. Due to the subtlety of mild DR, we grouped mild DR and non-DR (‘0’–‘1’) as normal, ‘2’–‘4’ as diseased and ‘5’ as strongly artefactual. We selected 50 high-quality images from both the normal and diseased classes. In addition, we selected 50 low-quality images from each of the normal and diseased classes to construct the weak artefact dataset, and 100 images from class ‘5’ to represent the strong artefact dataset.

### Metrics for model robustness

We manually categorised VLM responses into four classes: ‘0’ for those indicating normal findings without lesions, ‘1’ for responses indicating image abnormalities, ‘2’ for any cases where VLMs flag poor image quality and ‘3’ for instances where VLMs refused to answer due to ethical concerns or suggested consulting a specialist. All results and manual annotations are provided in Supplementary Data 4. We used three metrics to evaluate VLM robustness.

#### Model performance

The accuracy and sensitivity of VLMs in disease detection. Some responses from VLM did not provide a specific answer between disease and normal (with answers ‘2’ and ‘3’). We processed them as incorrect answers in calculating accuracy and sensitivity.

#### Performance percentage change

The performance difference between detecting diseases on images with weak artefacts and on original unaltered images, divided by original performance. We use relative performance change rather than the absolute difference to normalise performance changes against the baseline and make results comparable across tasks with different baseline accuracies. For example, suppose GPT-4o’s accuracy in detecting brain tumours on MRI scans before and after adding weak noise are 0.7 and 0.5, respectively, the performance percentage change is: (0.5–0.7)/0.7 = -28.6%.

#### Strong artefacts detection rate

The proportion of responses that flagged poor image quality in images with strong artefacts (with answer ‘2’), compared to the total number of responses.

### Models evaluated

This included three proprietary models—GPT-4o (2024.8.6)^2^, Claude 3.5 Sonnet (2024.6.20)^3^ and the upgraded version Claude 3.5 Sonnet (2024.10.22). Claude 3.5 Sonnet was upgraded during the experiment; we hence tested the performance of both versions. Additionally, we tested three open-source models, Llama 3.2 with 11 billion parameters^35^, BiomedCLIP^7^ and MedGemma^36^. BiomedCLIP and MedGemma are both designed for medical applications; however, BiomedCLIP provides only probability scores for each category in disease detection tasks. The chosen VLMs were selected for their popularity and strong performance across benchmarks based on previous literature^36,60,61^. Additionally, they have good practicality for clinicians and researchers to readily deploy and evaluate without extensive setting up and resource requirement. To maintain reproducibility, avoiding different responses for the same disease detection task, we set the temperature parameter as 0 for GPT-4o and both versions of Claude 3.5 Sonnet. For Llama 3.2, the temperature was configured to 0.01 to avoid the error that occurs when it is set to 0. BiomedCLIP and MedGemma do not define temperature parameters. The results and analysis in this study are fully reproducible. Although some proprietary VLMs didn’t release the details about their training data and architectures, our open-source code to generate synthetic images and assess the robustness of VLMs is available at: https://github.com/ziijiecheng/VLM_robustness. Additionally, all the benchmark data used for analysis in this study can be accessed at: https://drive.google.com/drive/folders/1M7EldoSvxEMZ2jA9wJs52H1-4G2zTU8C. As our study interprets VLM robustness to image artefacts based on VLM outputs, the study and its findings are reproducible and interpretable.

We tested three classical prompt strategies given that prompt substantially impacts VLMs’ performance: (1) structured output prompt, restricting the model to output only the categorical answer (e.g. ‘diseased’ or ‘normal’); (2) standard prompt, a default prompt style allowing open answering without special restrictions; and (3) Chain of Thought (CoT) prompt^44^, including instructions such as ‘Describe your reasoning step by step’ to encourage detailed, sequential reasoning. The details of these prompt strategies are put in the Supplementary Data 5. The three prompt strategies fit into diverse real-world healthcare scenarios. The structured output prompt facilitates efficient community screening where VLMs provide quick decisions and triage flags for large-scale cohorts. By contrast, the standard prompt and chain of thoughts can provide more evidence to support clinical decision-making. In particular, the chain of thought offers step-wide reasoning which may give descriptions of abnormal patterns such as lesion on medical images for disease detection.

### Statistical analysis

We employed stratified bootstrapping with 1000 iterations to maintain class distributions. We calculated the mean and standard deviation of the performance over the 1000 iterations and the standard error by (standard deviation/5). We obtain the 95% CI by means of 1.96 × standard error. To assess the statistical significance of performance changes due to adding artefacts, we conducted the two-tailed *t*-test to calculate *p* values and put the result in Supplementary Data 9.

## Supplementary information

supplementary material

Supplementary Data

## Data Availability

The benchmark dataset, which includes three imaging modalities with introduced artefacts, is derived from publicly available sources and can be accessed via the following links: MRI (https://www.kaggle.com/datasets/masoudnickparvar/brain-tumor-mri-dataset), OCT (https://www.kaggle.com/datasets/paultimothymooney/kermany2018) and X-ray (https://www.kaggle.com/datasets/darshan1504/covid19-detection-xray-dataset). We have reorganised, randomly sampled and added artefacts to create this benchmark, which is available at: https://github.com/ziijiecheng/VLM_robustness. Additionally, the colour fundus dataset, containing real-world artefacts, is available at: https://github.com/nkicsl/DDR-dataset. We have reorganised and sampled it according to image distortion levels, and it can be found alongside the benchmark dataset.

## Code availability

The code used to introduce artefacts and evaluate the performance of VLMs from Z.C. is available at: https://github.com/ziijiecheng/VLM_robustness. The code for introducing image artefacts uses TorchIO as the reference: https://github.com/TorchIO-project/torchio. The reference code for VLM evaluation is available for the following models: GPT-4o (https://platform.openai.com/docs/quickstart), Claude 3.5 Sonnet (https://github.com/anthropics/anthropic-cookbook), BiomedCLIP (https://huggingface.co/microsoft/BiomedCLIP-PubMedBERT_256-vit_base_patch16_224), Llama 3.2 (https://huggingface.co/meta-llama/Llama-3.2-11B-Vision-Instruct) and MedGemma (https://huggingface.co/google/medgemma-4b-pt). Results were further analysed and visualised using Python v.3.9.18, NumPy v.1.26.2, Matplotlib v.3.8.0, SciPy v.1.11.3 and pandas v.1.5.0.
