# Supplementary material for: Understanding the robustness of vision-language models to medical image artefacts
Source: NPJ Digit Med. 2025 Nov 27;8:727. doi: 10.1038/s41746-025-02108-w (PMC12660864; doi:10.1038/s41746-025-02108-w)
Supplement: Supplementary file 1 — supplementary material [file 41746_2025_2108_MOESM1_ESM.pdf]

Supplementary Material

Supplementary Fig. 1	MRI with artefacts
Supplementary Fig. 2	OCT with artefacts
Supplementary Fig. 3	X-ray with artefacts
Supplementary Fig. 4	VLMs' sensitivity original images
Supplementary Fig. 5	VLMs' specificity original images
Supplementary Fig. 6	VLMs' performance in MRI with weak artefacts
Supplementary Fig. 7	VLMs' performance in OCT with weak artefacts
Supplementary Fig. 8	VLMs' performance in X-ray with weak artefacts
Supplementary Fig. 9	VLMs' performance percentage change in MRI with the structured output prompt
Supplementary Fig. 10	VLMs' performance percentage change in MRI with the standard prompt
Supplementary Fig. 11	VLMs' performance percentage change in MRI with the Chain of Thought prompt
Supplementary Fig. 12	VLMs' performance percentage change in OCT with the structured output prompt
Supplementary Fig. 13	VLMs' performance percentage change in OCT with the standard prompt
Supplementary Fig. 14	VLMs' performance percentage change in OCT with the Chain of Thought prompt
Supplementary Fig. 15	VLMs' performance percentage change in X-ray with the structured output prompt
Supplementary Fig. 16	VLMs' performance percentage change in X-ray with the standard prompt
Supplementary Fig. 17	VLMs' performance percentage change in X-ray with the Chain of Thought prompt
Supplementary Fig. 18	VLMs’ robustness in colour fundus photographs with real world artefact
Supplementary Fig. 19	MedGemma’s conflict rate
Supplementary Fig. 20	Examples of color fundus images with real-world image artefacts from DDR datasets
Supplementary Fig. 21	An example of weak random cropping on a brain MRI image
Supplementary Data 1	False positive/negative examples
Supplementary Data 2	Benchmark summary
Supplementary Data 3	Image artefacts settings
Supplementary Data 4	All VLMs' responses
Supplementary Data 5	Prompts' detail
Supplementary Data 6	Quantitative results of VLMs' original performance
Supplementary Data 7	Quantitative results of VLMs' performance after adding weak artefacts
Supplementary Data 8	Quantitative results of VLMs' performance percentage change after adding weak artefacts
Supplementary Data 9	p-values for model performance at different scales of image artefacts
Supplementary Data 10	Quantitative results of VLMs' strong artefacts detection rate
Supplementary Data 11	Quantitative results of VLMs’ robustness in colour fundus photographs with real world artefact
Supplementary Data 12	Quantitative results of MedGemma’s conflict rate
Supplementary Data 13	An example of MedGemma’ s reasoning process conflict with its final conclusion

Supplementary Figures

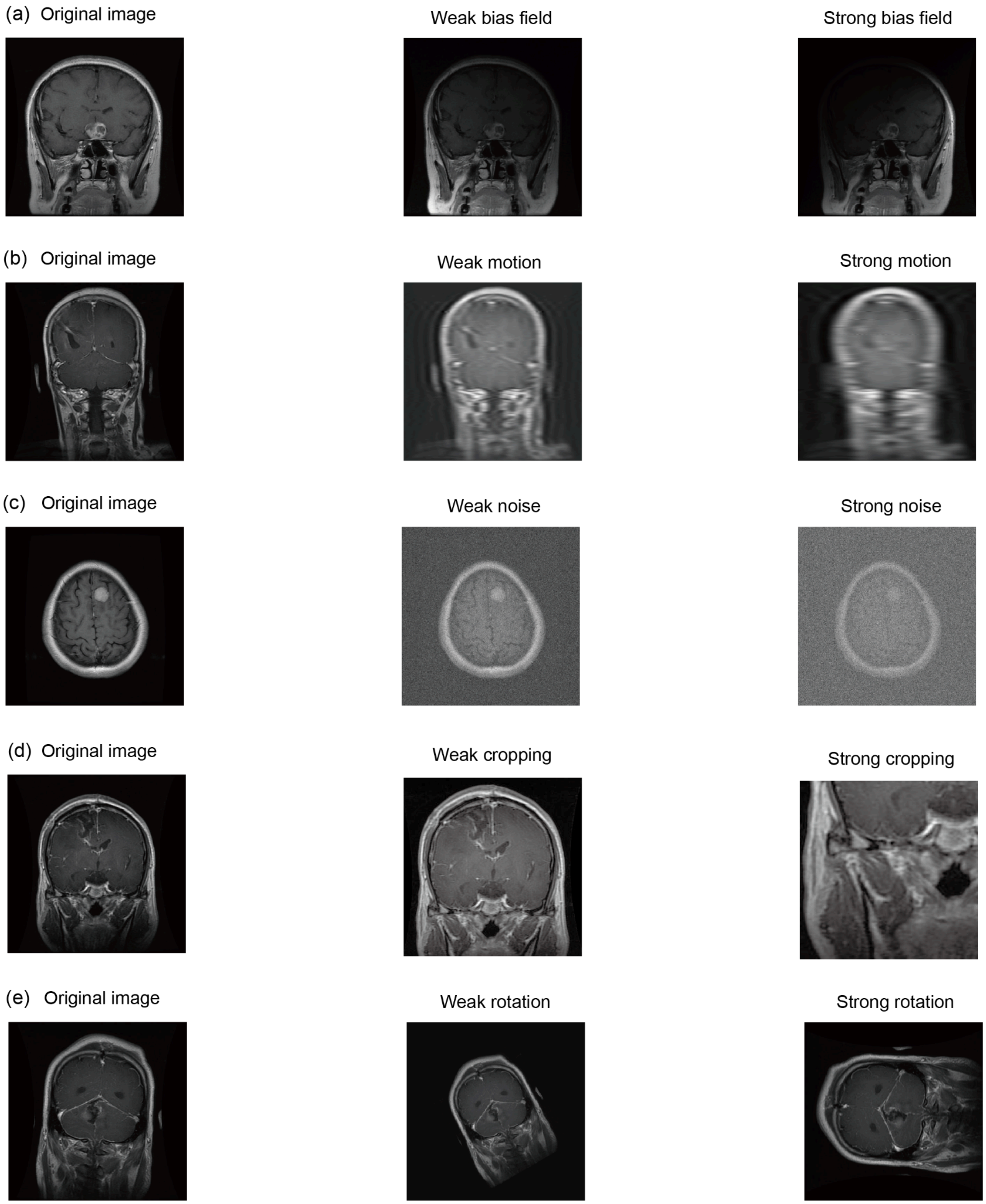

Supplementary Fig. 1: Examples of brain MRI images with weak and strong artefacts (a)~(e).

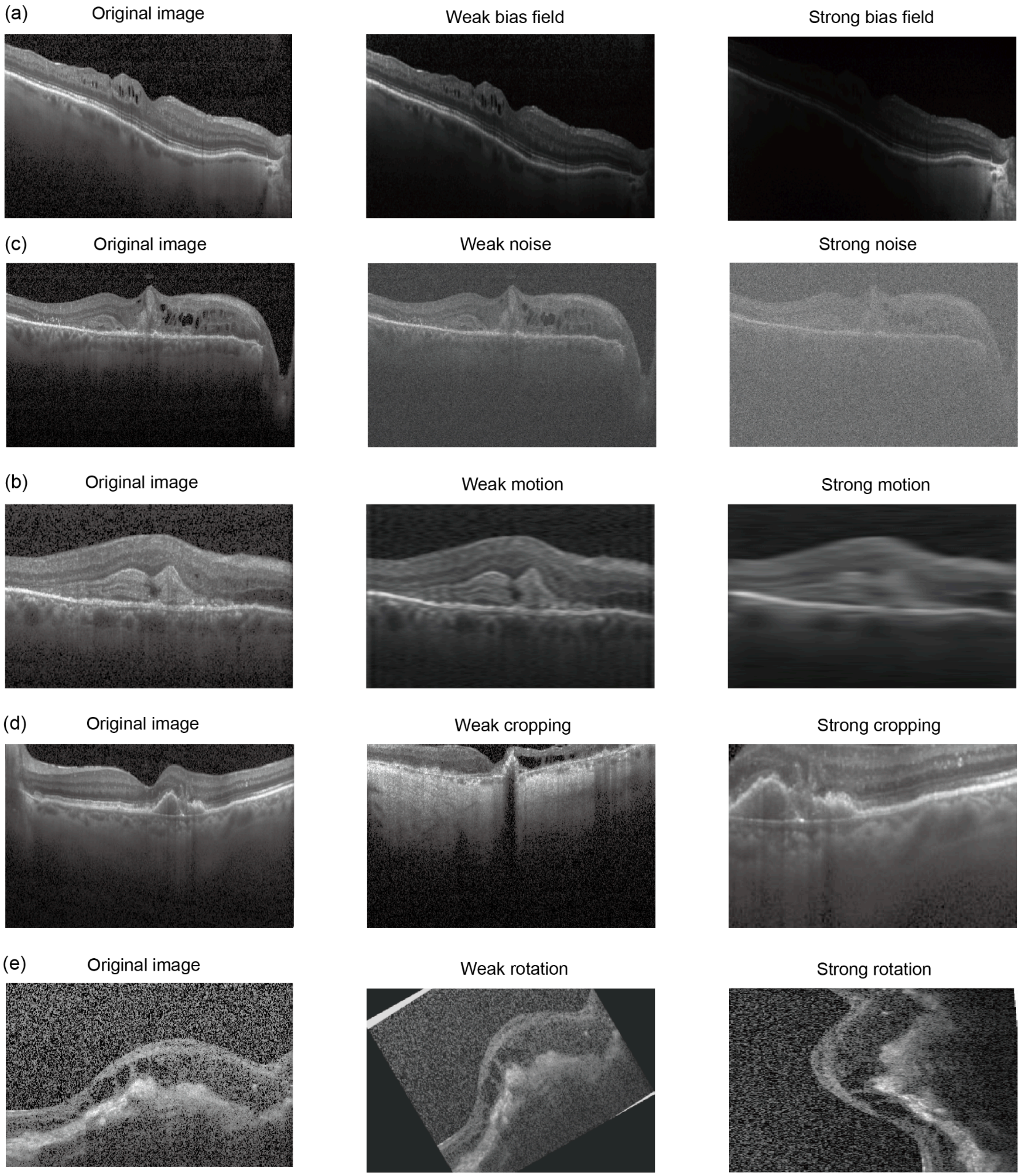

Supplementary Fig. 2: Examples of OCT images with weak and strong artefacts (a)~(e).

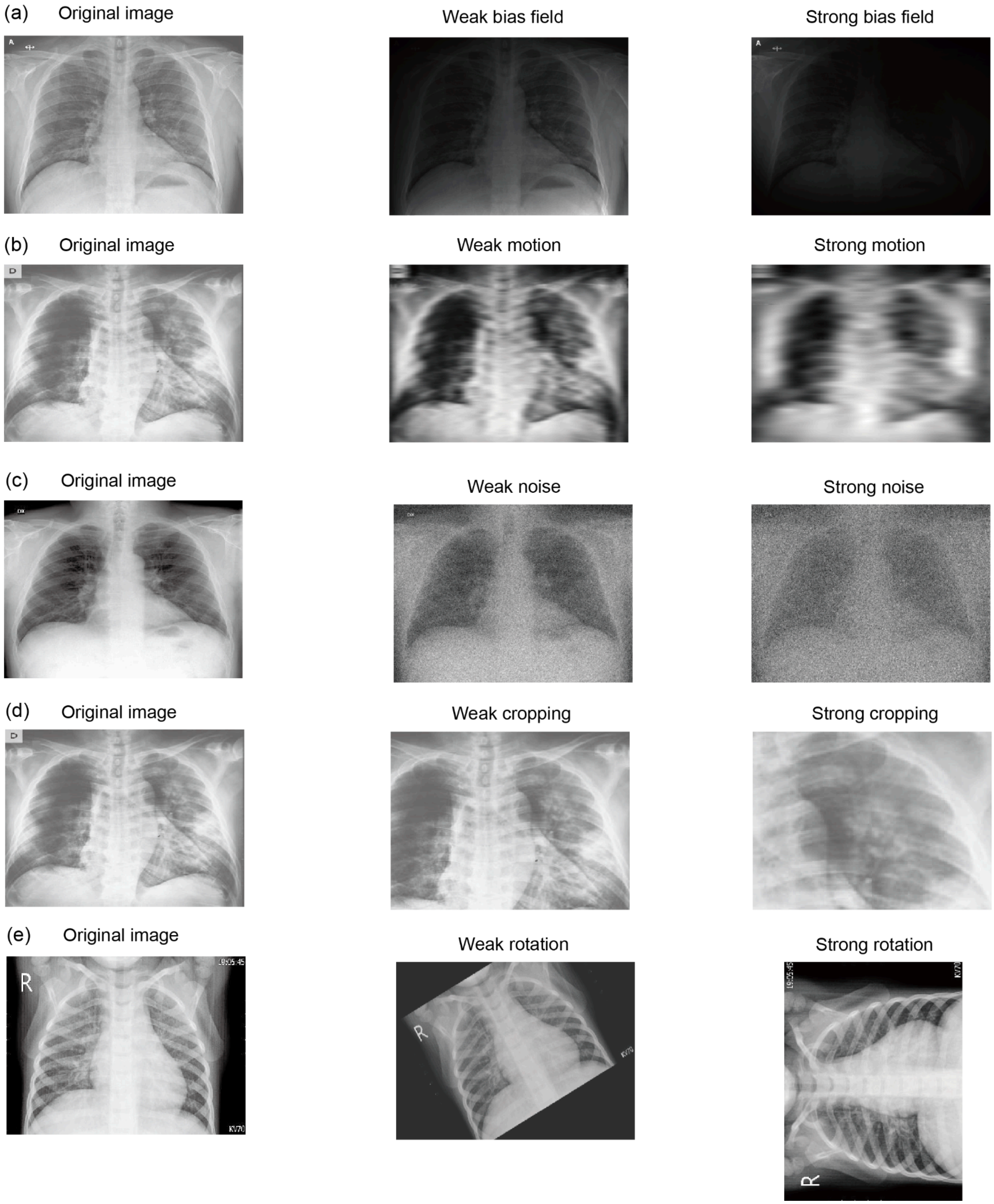

Supplementary Fig. 3: Examples of chest X-ray with weak and strong artefacts (a)~(e).

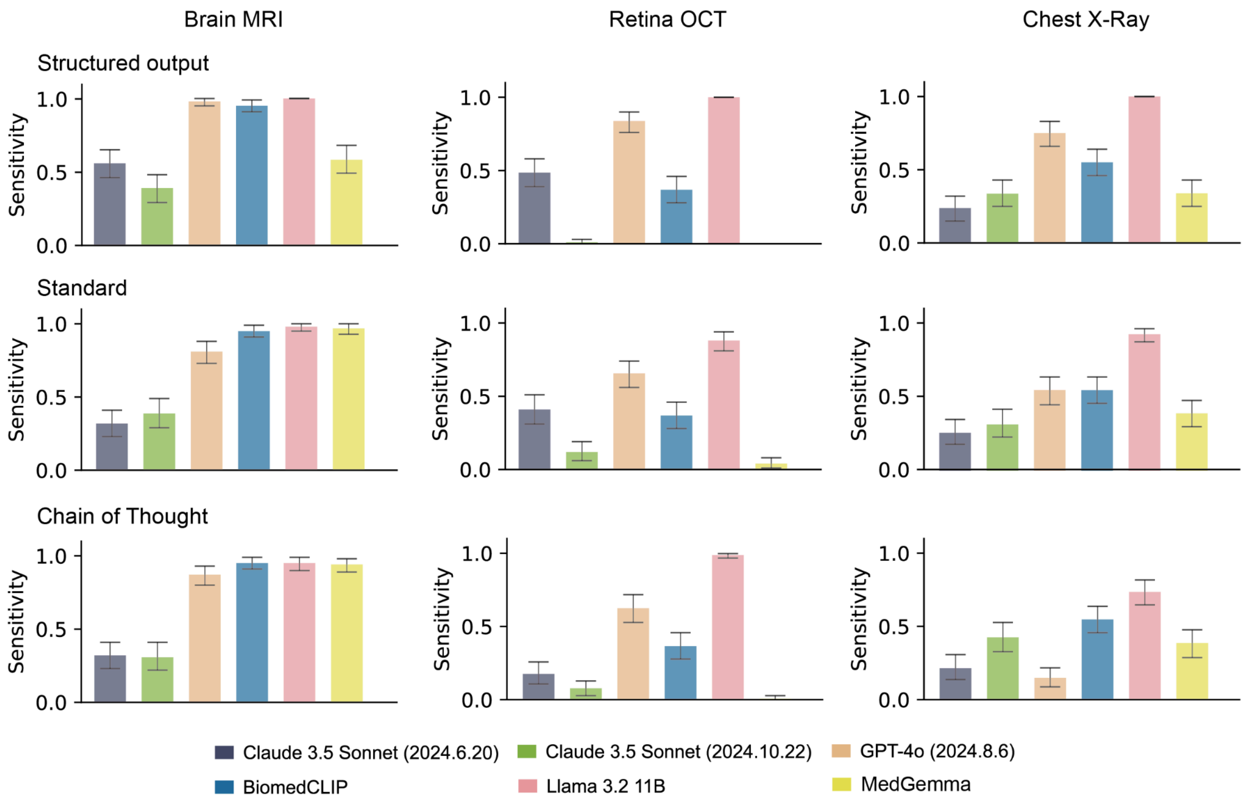

Supplementary Fig. 4: The sensitivity of Vision-Language models (VLMs) in detecting disease from original unaltered images. Each column represents the models' sensitivity in disease detection tasks across different medical imaging modalities, while each row illustrates their accuracy using various prompt strategies. Quantitative results are detailed in Supplementary Data 6. For each task, sensitivity measurements were derived from 1000 stratified bootstrap samples. Results show mean sensitivity with 95% confidence intervals (error bars).

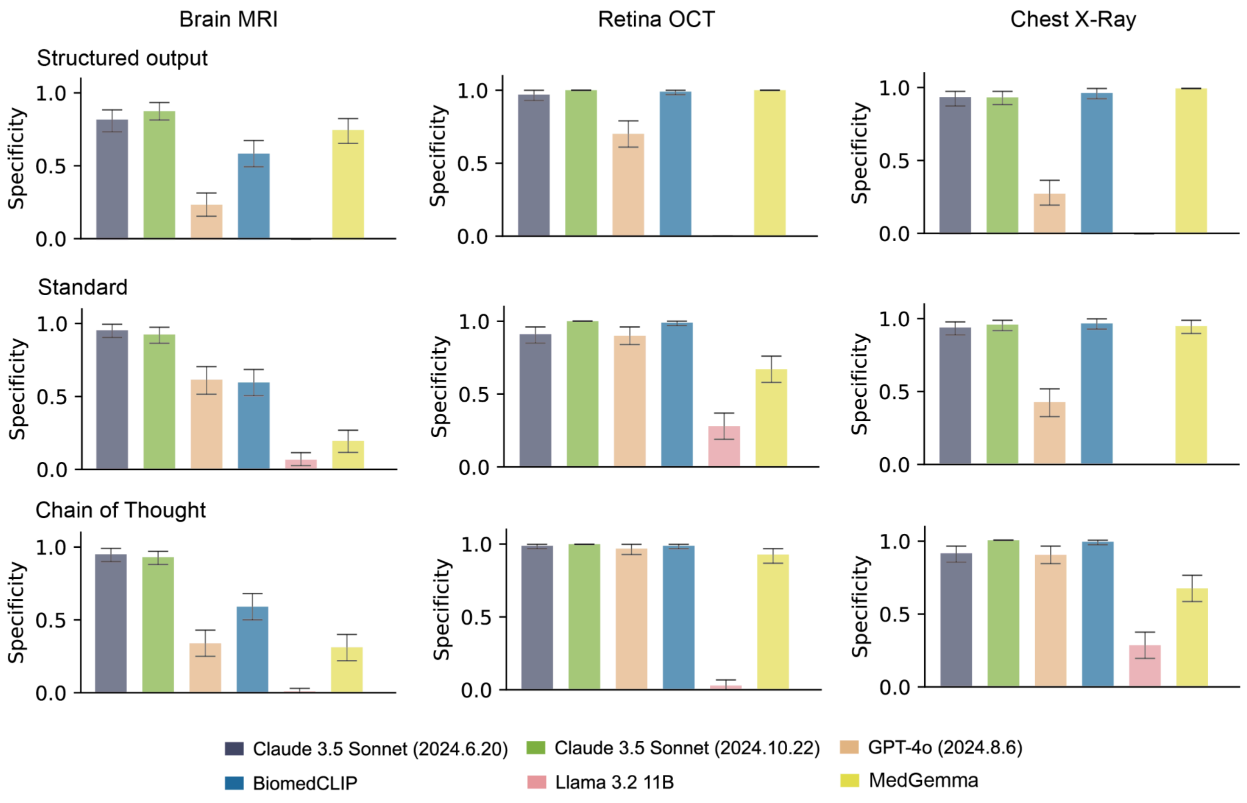

Supplementary Fig. 5: The specificity of Vision-Language models (VLMs) in detecting disease from original unaltered images. Each column represents the models' specificity in disease detection tasks across different medical imaging modalities, while each row illustrates their accuracy using various prompt strategies. Quantitative results are detailed in Supplementary Data 6. For each task, specificity measurements were derived from 1000 stratified bootstrap samples. Results show mean specificity with 95% confidence intervals (error bars).

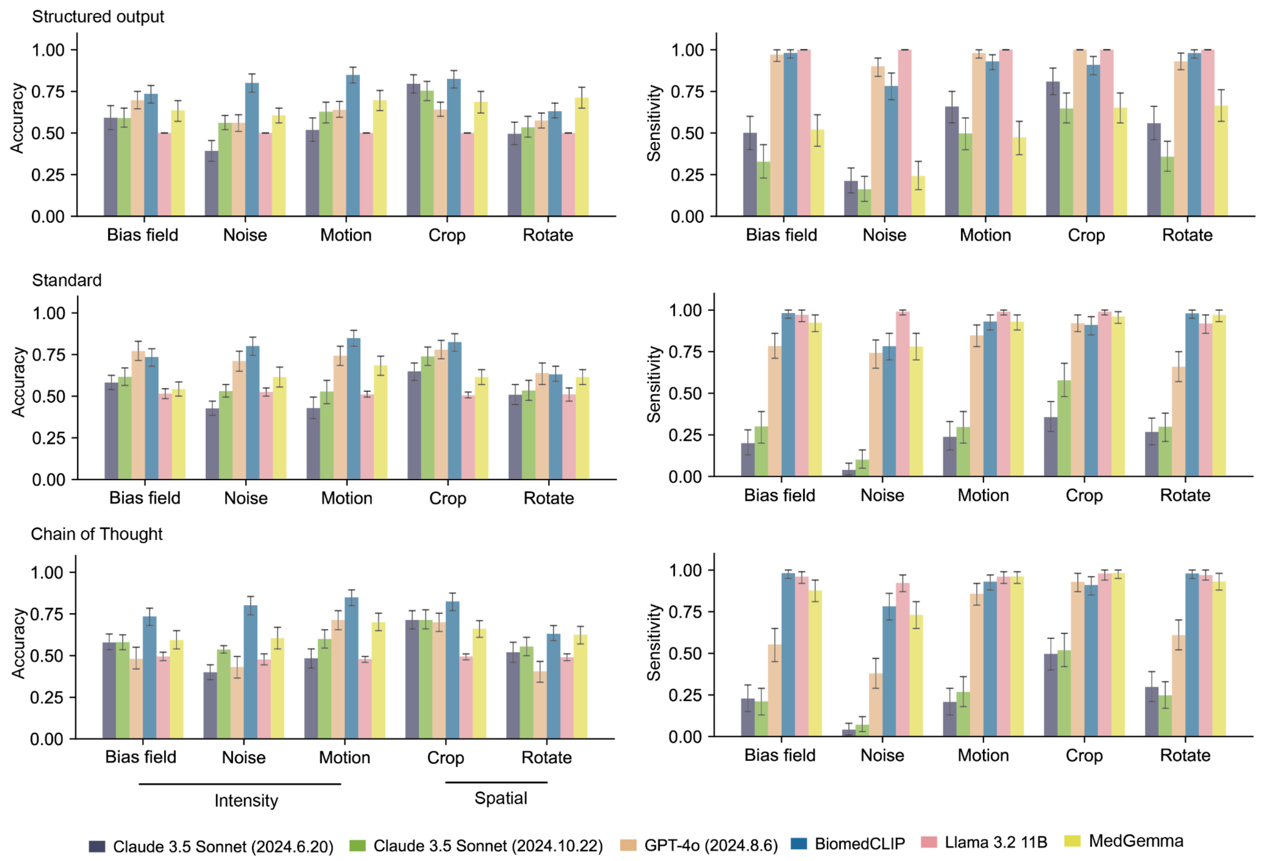

Supplementary Fig. 6: The model performance of all Vision-Language models (VLMs) in MRI applications after adding weak artefacts to the original unaltered images. Each row represents the models' accuracy and sensitivity in disease detection tasks, while each column shows how performance varies with different prompt strategies. Complete quantitative results for VLMs' accuracy, sensitivity in brain tumour detection are available in Supplementary Data 7. For each disease detection task, we performed 1,000 iterations of stratified bootstrapping to calculate accuracy and sensitivity. Results show mean performance with 95% confidence intervals (error bars).

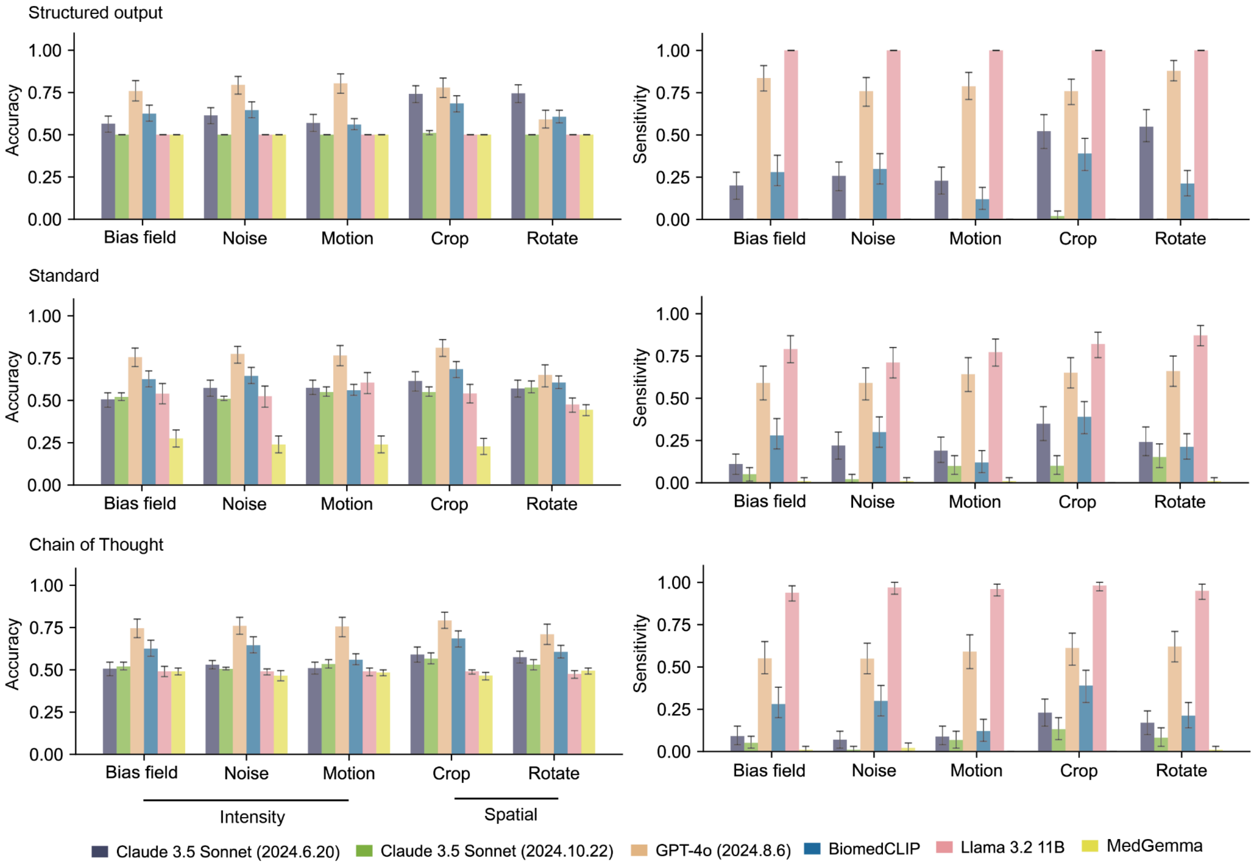

Supplementary Fig. 7: The model performance of all Vision-Language models (VLMs) in OCT applications after adding weak artefacts to the original unaltered images. Each row represents the models' accuracy and sensitivity in disease detection tasks, while each column shows how performance varies with different prompt strategies. Complete quantitative results for VLMs' accuracy and sensitivity in macular disease detection are available in Supplementary Data 7. For each task, we performed 1,000 iterations of stratified bootstrapping to calculate accuracy and sensitivity. Results show mean performance with 95% confidence intervals (error bars).

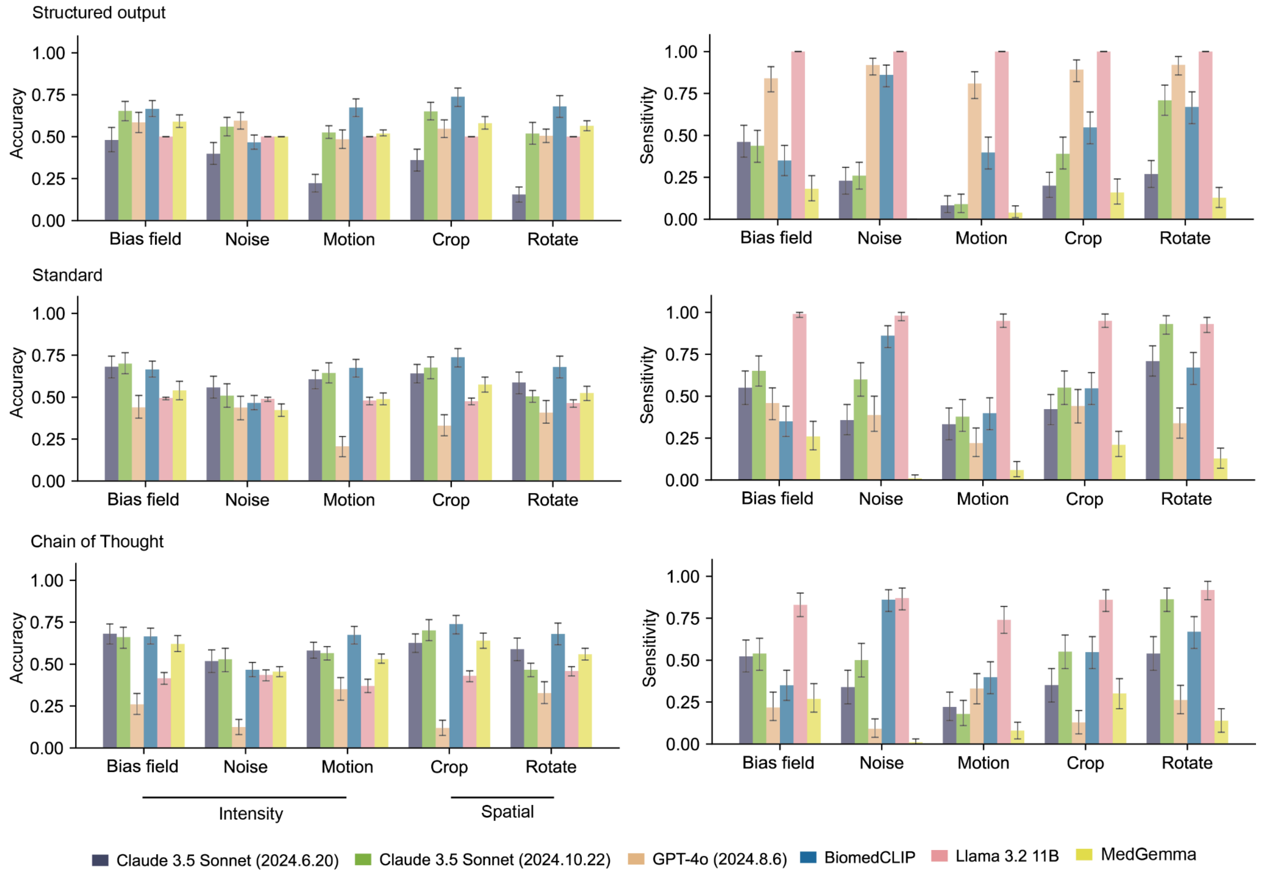

Supplementary Fig. 8: The model performance of all Vision-Language models (VLMs) in X-ray applications after adding weak artefacts to the original unaltered images. Each row represents the models' accuracy and sensitivity in disease detection tasks, while each column shows how performance varies with different prompt strategies. Complete quantitative results for VLMs' accuracy, sensitivity in Covid/pneumonia detection are available in Supplementary Data 7. For each task, we performed 1,000 iterations of stratified bootstrapping to calculate accuracy and sensitivity. Results show mean performance with 95% confidence intervals (error bars).

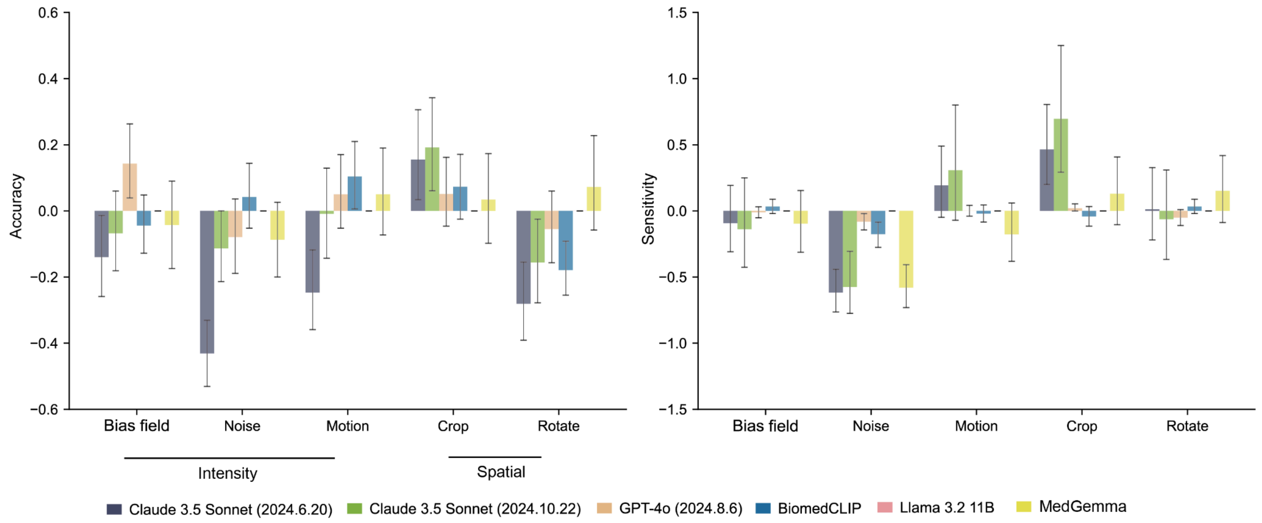

Supplementary Fig. 9: Performance percentage change of all VLMs after adding weak artefacts to original MRI images, assessed with the structured output prompt. The y-axis represents the percentage change in performance, with positive values indicating increased performance and negative values indicating a decrease. Complete quantitative results are available in Supplementary Data 8. For each task, we calculated performance percentage change through 1,000 iterations of stratified bootstrapping. Results show mean performance with 95% confidence intervals (error bars).

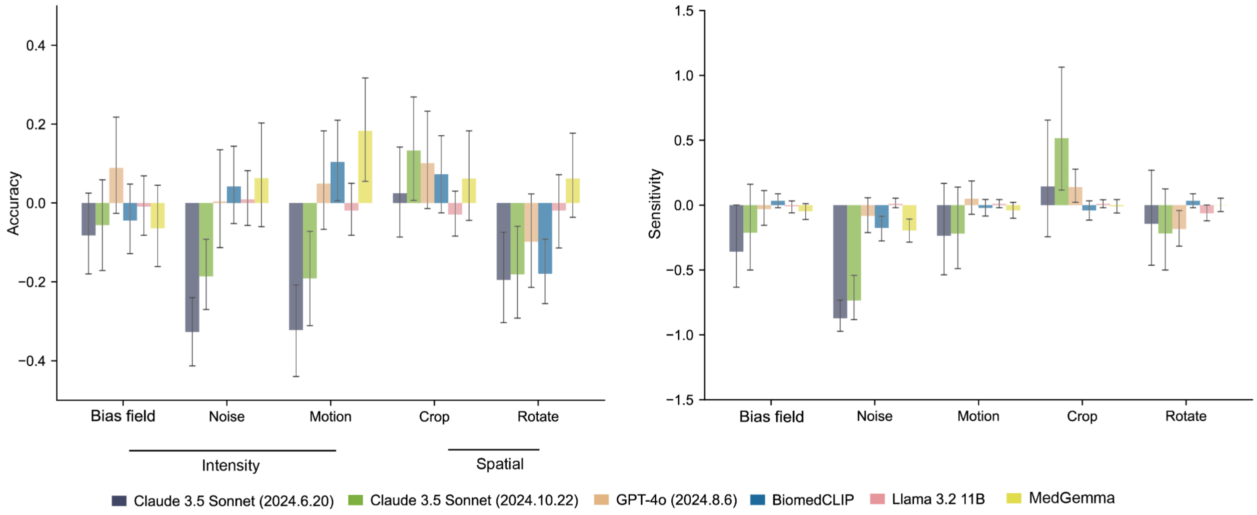

Supplementary Fig. 10: Performance percentage change of all VLMs after adding weak artefacts to original MRI images, assessed with standard prompts. The y-axis represents the percentage change in performance, with positive values indicating increased performance and negative values indicating a decrease. Complete quantitative results are available in Supplementary Data 8. For each task, we calculated performance percentage change through 1,000 iterations of stratified bootstrapping. Results show mean performance with 95% confidence intervals (error bars).

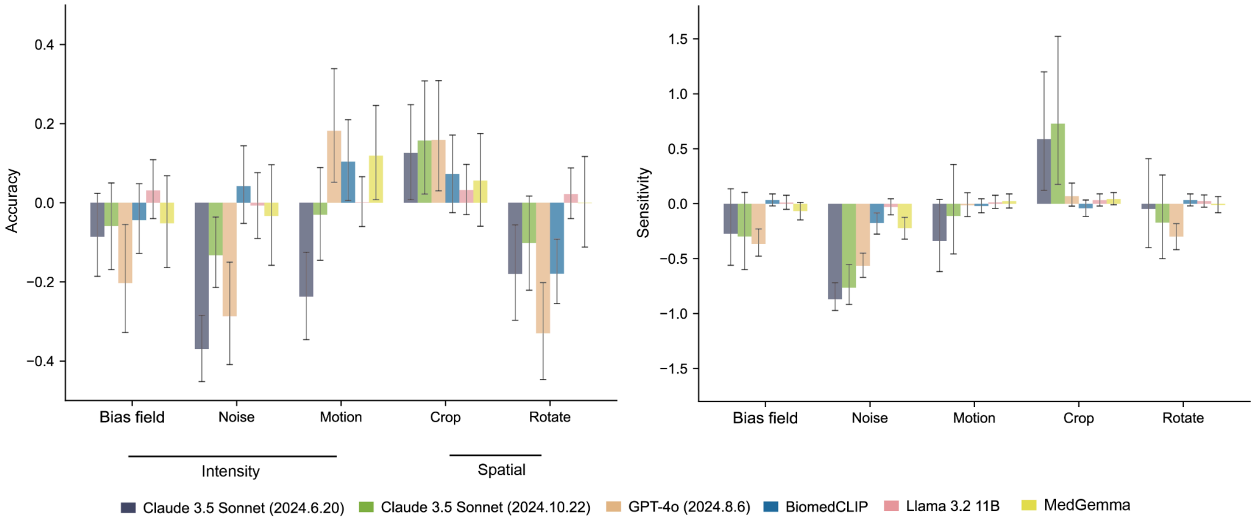

Supplementary Fig. 11: Performance percentage change of all VLMs after adding weak artefacts to original MRI images, assessed with Chain of Thought prompts. The y-axis represents the percentage change in performance, with positive values indicating increased performance and negative values indicating a decrease. Complete quantitative results are available in Supplementary Data 8. For each task, we calculated performance percentage change through 1,000 iterations of stratified bootstrapping. Results show mean performance with 95% confidence intervals (error bars).

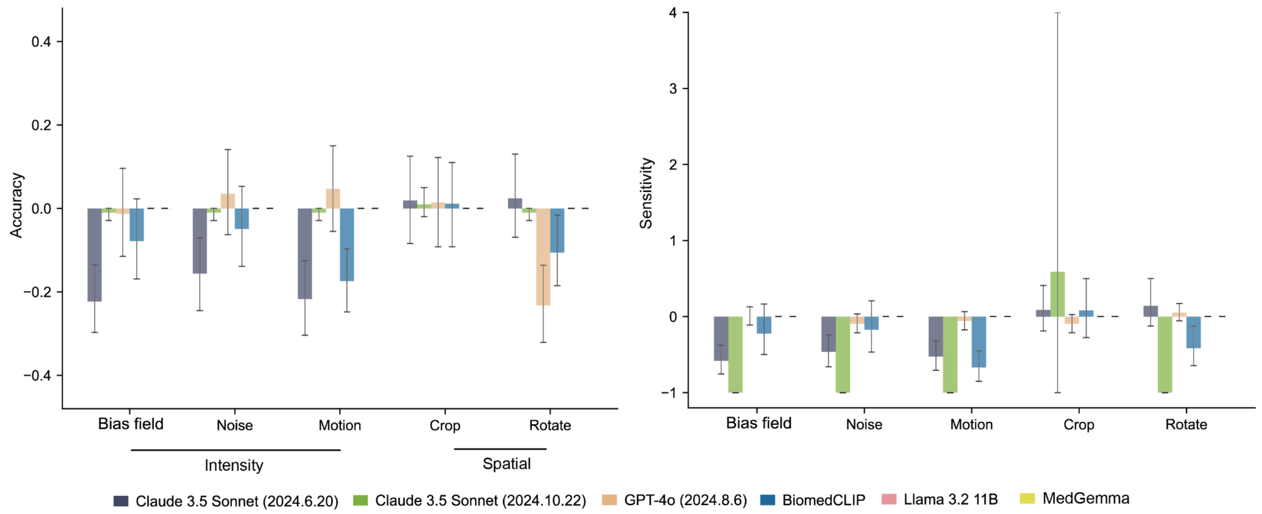

Supplementary Fig. 12: Performance percentage change of all VLMs after adding weak artefacts to original OCT images, assessed with structured output prompts. The y-axis represents the percentage change in performance, with positive values indicating increased performance and negative values indicating a decrease. Complete quantitative results are available in Supplementary Data 8. For each task, we calculated performance percentage change through 1,000 iterations of stratified bootstrapping. Results show mean performance with 95% confidence intervals (error bars).

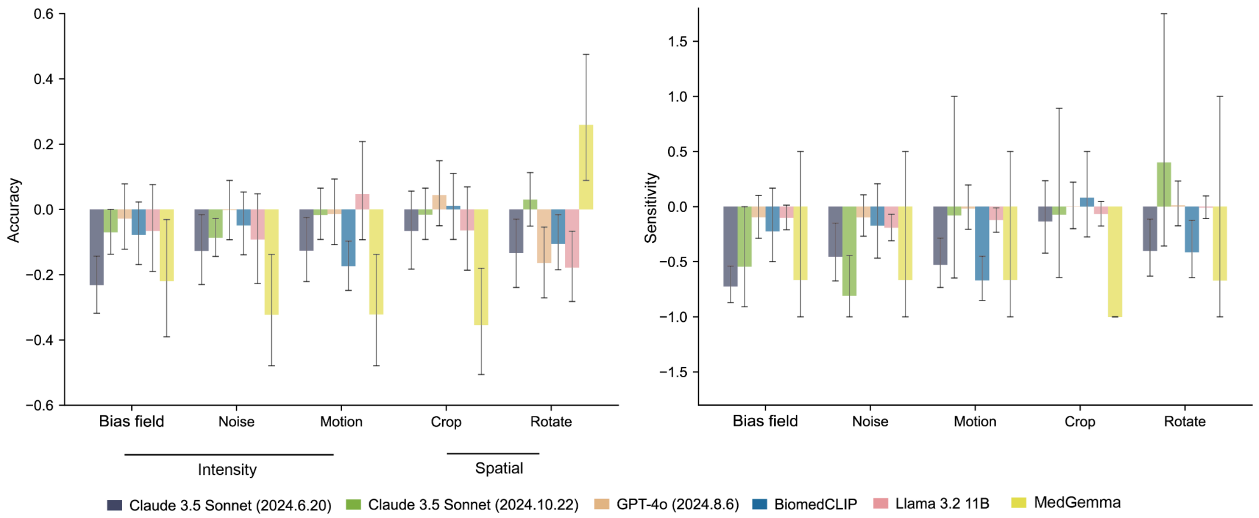

Supplementary Fig. 13: Performance percentage change of all VLMs after adding weak artefacts to original OCT images, assessed with standard prompts. The y-axis represents the percentage change in performance, with positive values indicating increased performance and negative values indicating a decrease. Complete quantitative results are available in Supplementary Data 8. For each task, we calculated performance percentage change through 1,000 iterations of stratified bootstrapping. Results show mean performance with 95% confidence intervals (error bars).

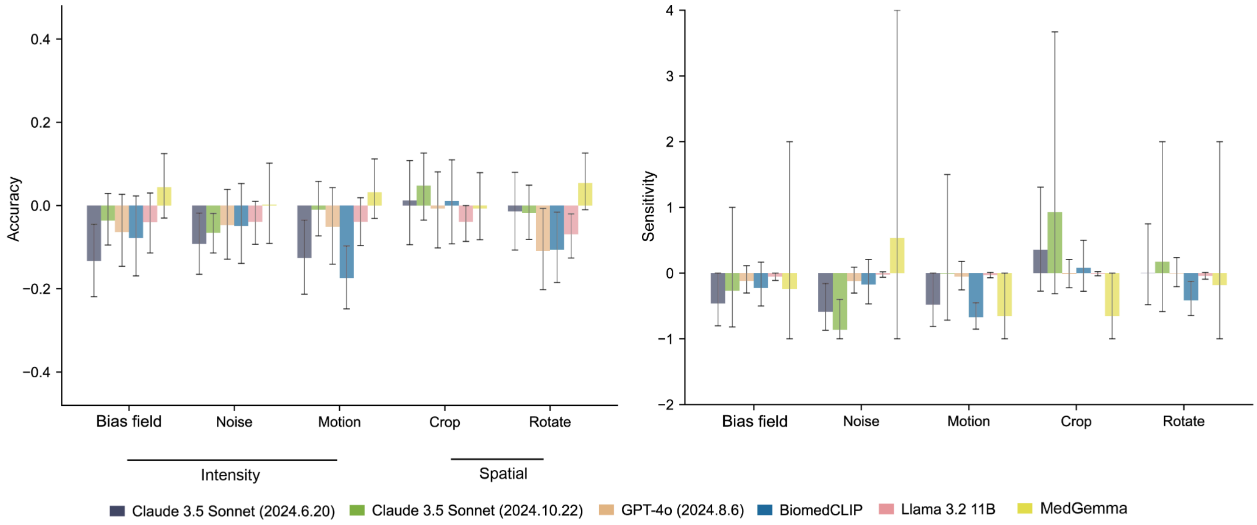

Supplementary Fig. 14: Performance percentage change of all VLMs after adding weak artefacts to original OCT images, assessed with Chain of Thought prompts. The y-axis represents the percentage change in performance, with positive values indicating increased performance and negative values indicating a decrease. Complete quantitative results are available in Supplementary Data 8. For each task, we calculated performance percentage change through 1,000 iterations of stratified bootstrapping. Results show mean performance with 95% confidence intervals (error bars).

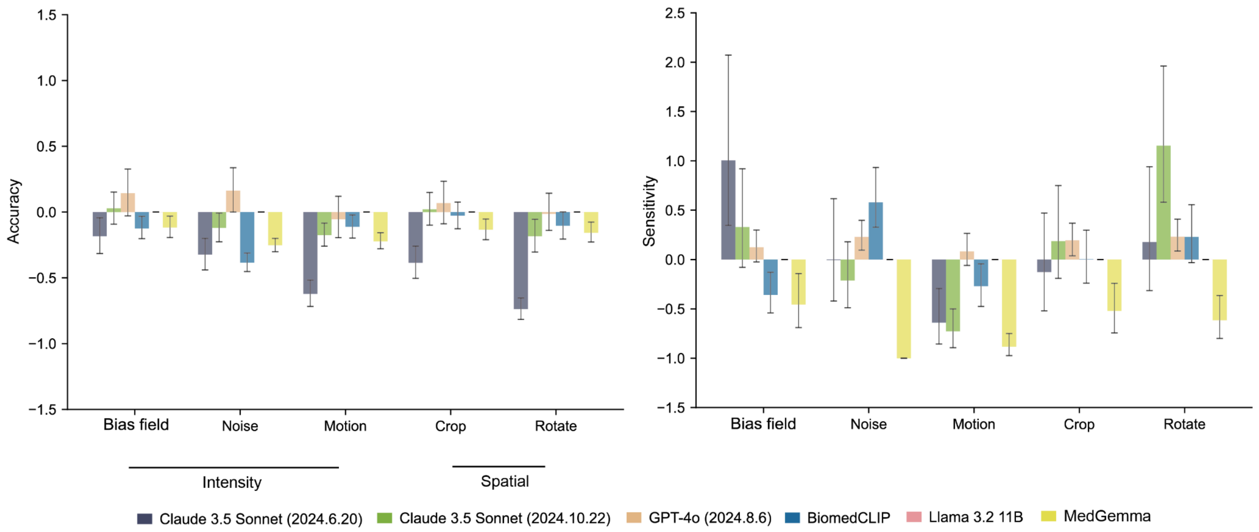

Supplementary Fig. 15: Performance percentage change of all VLMs after adding weak artefacts to original X-ray images, assessed with structured output prompts. The y-axis represents the percentage change in performance, with positive values indicating increased performance and negative values indicating a decrease. Complete quantitative results are available in Supplementary Data 8. For each task, we calculated performance percentage change through 1,000 iterations of stratified bootstrapping. Results show mean performance with 95% confidence intervals (error bars).

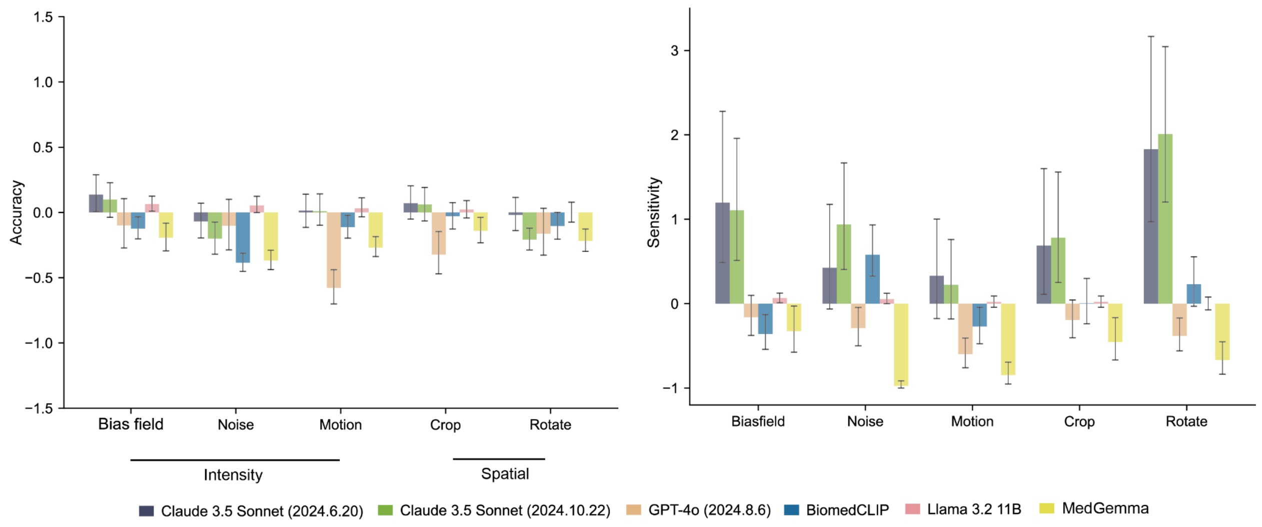

Supplementary Fig. 16: Performance percentage change of all VLMs after adding weak artefacts to original X-ray images, assessed with standard prompts. The y-axis represents the percentage change in performance, with positive values indicating increased performance and negative values indicating a decrease. Complete quantitative results are available in Supplementary Data 8. For each task, we calculated performance percentage change through 1,000 iterations of stratified bootstrapping. Results show mean performance with 95% confidence intervals (error bars).

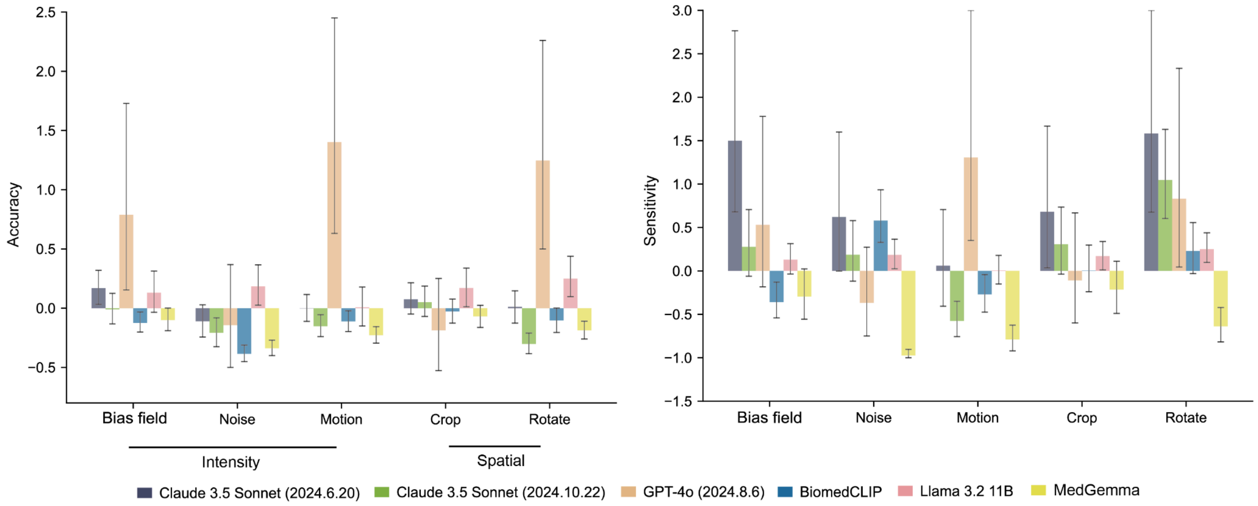

Supplementary Fig. 17: Performance percentage change of all VLMs after adding weak artefacts to original X-ray images, assessed with Chain-of-Thought prompts. The y-axis represents the percentage change in performance, with positive values indicating increased performance and negative values indicating a decrease. Complete quantitative results are available in Supplementary Data 8. For each task, we calculated performance percentage change through 1,000 iterations of stratified bootstrapping. Results show mean performance with 95% confidence intervals (error bars).

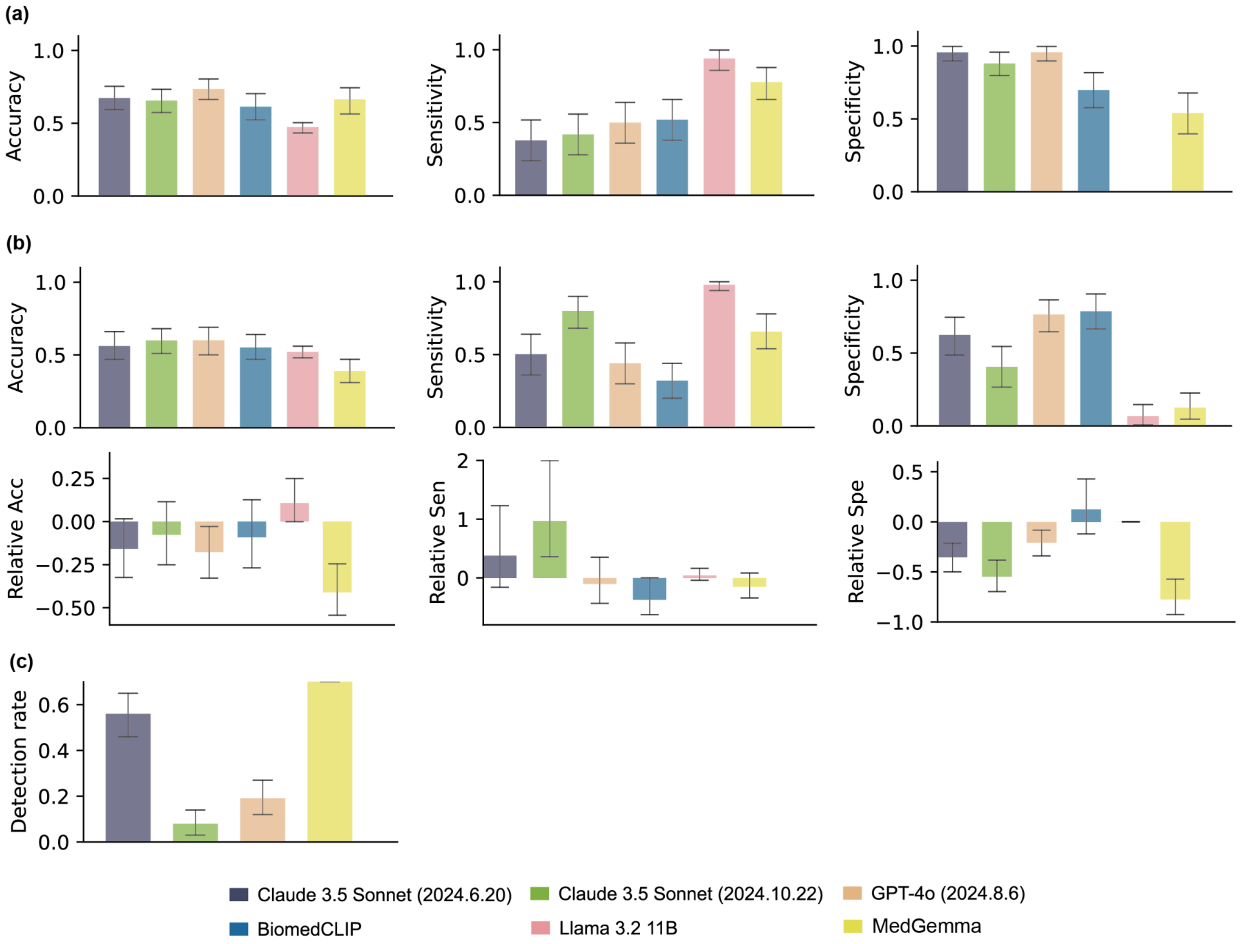

Supplementary Fig. 18: Robustness of Vision-Language Models (VLMs) in detecting diabetic retinopathy from color fundus images with real-world artefacts. Figure (a) illustrates the models' performance when evaluating high-quality color fundus images. Figure (b) presents the models' performance on images with weak artefacts, along with their performance percentage change compared to high-quality images. Figure (c) displays the VLMs' artefact detection rate when evaluating ungradable images. Quantitative results are provided in Supplementary Data 11. For each task, we performed 1,000 iterations of stratified bootstrapping to estimate the mean performance with 95% confidence intervals (error bars).

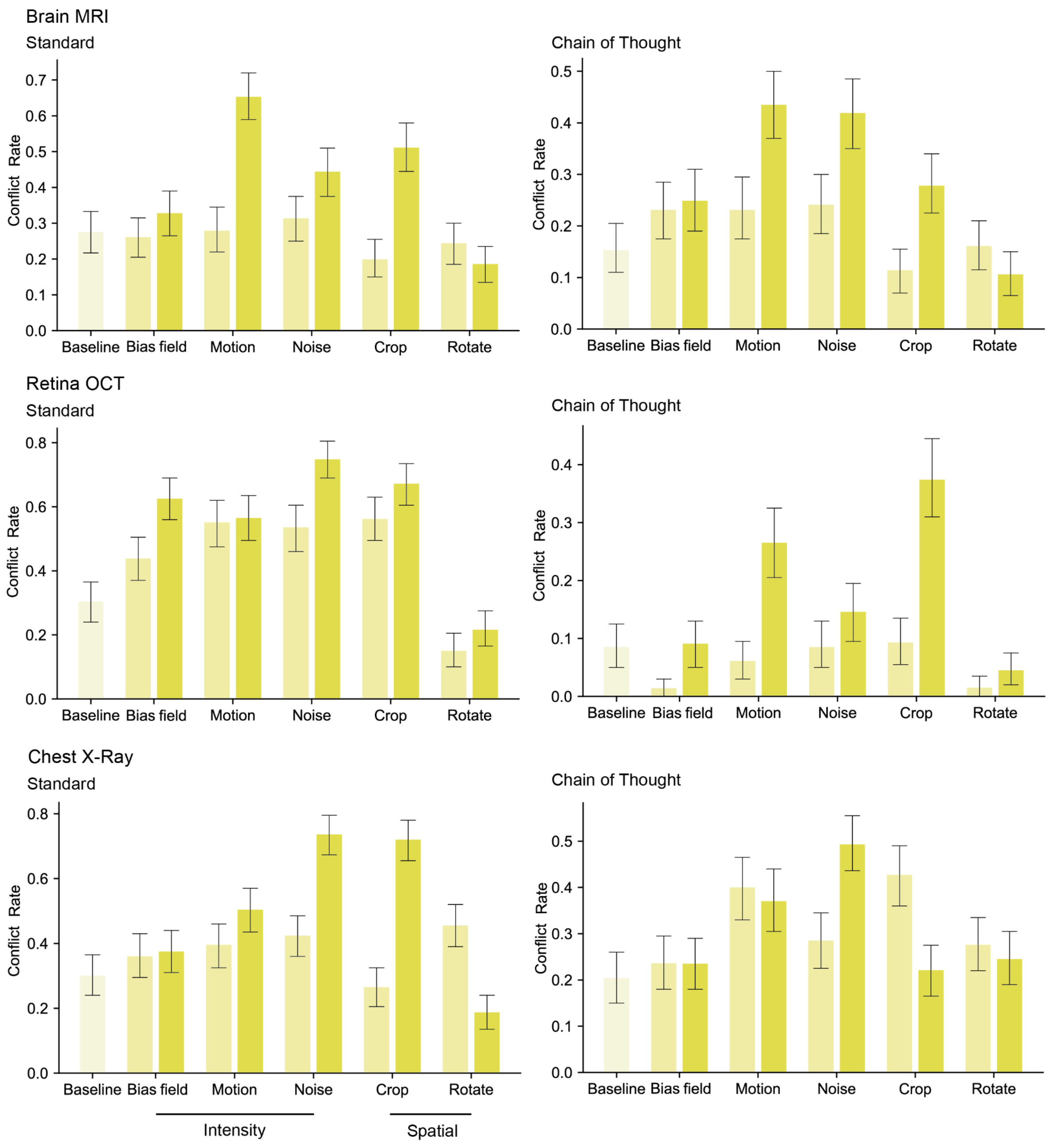

Supplementary Fig. 19: Rate of conflict between MedGemma's reasoning process and final conclusions. Each row shows the conflict rate in disease detection tasks across different medical imaging modalities, while each column shows the conflict rate across different prompt strategies. Each subfigure presents the conflict rate across various types and scales of artefacts, with darker yellow indicating stronger artefacts. Quantitative results are provided in Supplementary Data 12. For each task, we performed 1,000 iterations of stratified bootstrapping to estimate the mean performance with 95% confidence intervals (shown as error bar).

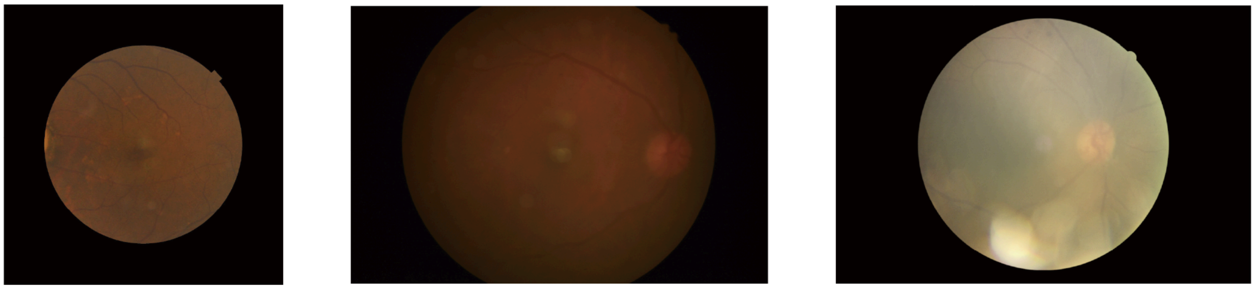

(1) (2) (3)

Supplementary Fig. 20: Examples of color fundus images with real-world image artefacts from DDR datasets (without synthetic artefacts). The first image (from left to right) exhibits random cropping (clearly smaller field of vision) in conjunction with random noise; the second image presents random noise combined with motion blur and the third image demonstrates both bias field artefacts and random noise. On the DDR dataset, we observed similar findings as what we observed on images with added single artefact, as illustrated in Figures 4 and Extended Data Figure 18. Specifically, VLMs show decreased performance on images containing single artefact and real-world compound artefacts compared to good quality images.

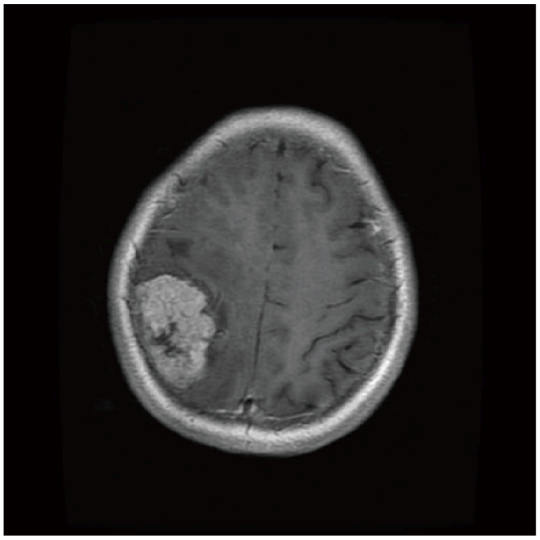

Original MRI image

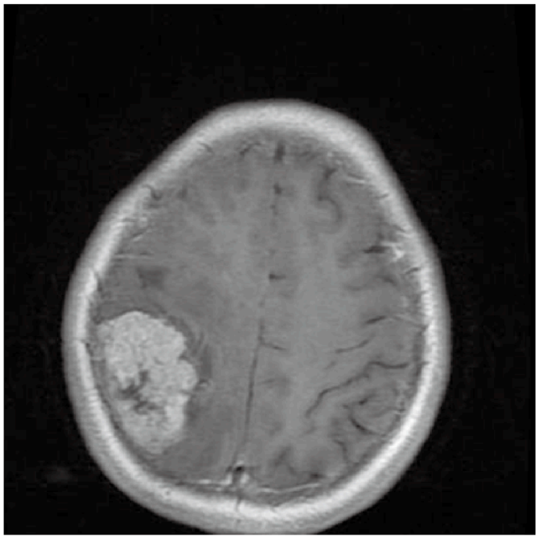

The Cropped MRI image

Supplementary Fig. 21: An example of weak random cropping on a brain MRI image. The left image is an original unaltered brain MRI image. The right image is the same image after adding weak random cropping.

Supplementary Data

Supplementary Data 1: False positive/negative examples. The table presents cases in which models accurately classify original unaltered images as either normal or diseased, but produce misclassifications when artefacts are introduced. Specifically, it reports five false-positive and five false-negative examples for three tasks: GPT-4o detecting brain tumours from MRI scans, GPT-4o detecting retinal diseases from OCT images, and Claude 3.5 Sonnet detecting lung diseases from chest X-rays. The first column specifies the image modality, the second column lists the image name, the third column presents the models' predictions on the original unaltered images, and the fourth column records their predictions after the introduction of artefacts.

Supplementary Data 2: Benchmark summary. The table presents the benchmark structure comprising images from three distinct modalities. For each modality, the dataset includes 200 images, consisting of 100 normal and 100 diseased cases. Five types of image artefacts are introduced into these images at both weak and strong scales.

Supplementary Data 3: Image artefacts settings. The table presents the image artefact settings (hyperparameters) used when applying bias fields, random noise, and motion artefacts from the TorchIO library, as well as the cropping proportions and rotation angles.

Supplementary Data 4: All VLMs' responses. The table presents the VLMs' responses in disease detection tasks across three medical image modalities. Each task was performed under three different prompt strategies. The table reports the names of the evaluated images, their corresponding ground-truth labels, the VLMs' predictions, and our manual assessment of those predictions (decision).

Supplementary Data 5: Prompts' detail. The table presents three prompt strategies, each applied to different models across various image modalities.

Supplementary Data 6: Quantitative results of VLMs' original performance. The table presents VLMs' accuracy, sensitivity and specificity in detecting diseases from original unaltered images. It also shows VLMs' performance under various prompt strategies. For each task, performance measurements derive from 1000 stratified bootstrap samples. Results show mean performance with 95% confidence intervals.

Supplementary Data 7: Quantitative results of VLMs' performance after adding weak artefacts. The table presents VLMs' accuracy, sensitivity and specificity in detecting diseases from images with added weak artefacts. It also shows VLMs' performance under various prompt strategies. For each task, performance measurements derive from 1000 stratified bootstrap samples. Results show mean performance with 95% confidence intervals.

Supplementary Data 8: Quantitative results of VLMs' performance percentage change after adding weak artefacts. The table presents the relative changes in accuracy and

sensitivity of VLMs in disease detection tasks when weak image artefacts are introduced, compared with performance on the original unaltered images. These percentage changes are further evaluated across a range of prompt strategies. For each task, performance change estimates were obtained from 1,000 stratified bootstrap samples. Results are presented as mean values with corresponding 95% confidence intervals.

Supplementary Data 9: P-values for model performance at different scales of image artefacts. The table presents p-values comparing VLMs' accuracy, sensitivity, and specificity in detecting lesions from original unaltered images versus images with weak artefacts. The p-values are tested through two-tailed t-test with 1,000 stratified bootstrap samples.

Supplementary Data 10: Quantitative results of VLMs' strong artefacts detection rate. The table presents VLMs' strong artefacts detection rate on severely distorted images. It also shows this performance under various prompt strategies. For each task, performance measurements derive from 1000 stratified bootstrap samples. Results show mean performance with 95% confidence intervals.

Supplementary Data 11: Quantitative results of VLMs' robustness in colour fundus photographs with real world artefact. The table presents VLMs' accuracy, sensitivity, and specificity in disease detection tasks using high-quality colour fundus photographs and photographs containing real-world weak artefacts. It also reports the percentage change in performance between high-quality images and those with real-world weak artefacts, as well as VLMs' strong artefacts detection rate in severely distorted real-world images. For each task, performance measurements derive from 1000 stratified bootstrap samples. Results show mean performance with 95% confidence intervals.

Supplementary Data 12: Quantitative results of MedGemma's conflict rate. The table presents the rate of conflict between VLMs' reasoning process and their conclusions. The conflict rate is evaluated when VLMs detect lesions from original unaltered images and from images with artefacts at both weak and strong scales. It also reports p-values for comparisons across different artefact scales, calculated using two-tailed t-tests with 1,000 stratified bootstrap samples. All measurements are repeated under three distinct prompt strategies.

Supplementary Data 13: An example of MedGemma' s reasoning process conflict with its final conclusion. For an image with weak artefacts, the intermediate analysis states that "the image is too low in resolution to adequately assess the disease features". However, the final conclusion said "Normal", conflicting with the reasoning process.
